# Pesticide exposures and chronic kidney disease of unknown etiology: an epidemiologic review

**DOI:** 10.1186/s12940-017-0254-0

**Published:** 2017-05-23

**Authors:** Mathieu Valcke, Marie-Eve Levasseur, Agnes Soares da Silva, Catharina Wesseling

**Affiliations:** 1 WHO-PAHO Collaborating Centre on Environmental and Occupational Health Impact Assessment and Surveillance INSPQ-CHUQ-DSPQ, 945, Avenue Wolfe, Québec, G1V 5B3 Canada; 2 Department of Environmental and Occupational Health, School of Public Health, Université de Montréal, C.P. 6128 Succursale Centre-Ville, Montreal, H3C 3J7 Canada; 3 Pan American Health Organization (PAHO), 525 Twenty-third Street, N.W, Washington DC, 20037 USA; 4 Department of Occupational Medicine, Institute of Environmental Medicine (IMM), Karolinska Institutet, 171 77 Stockholm, SE Sweden

**Keywords:** Agrochemicals, Chronic kidney disease of unknown etiology (CKDu), Etiology, Exposure, Pesticides, Review

## Abstract

The main causes of chronic kidney disease (CKD) globally are diabetes and hypertension but epidemics of chronic kidney disease of unknown etiology (CKDu) occur in Central America, Sri Lanka, India and beyond. Althoug also being observed in women, CKDu concentrates among men in agricultural sectors. Therefore, suspicions fell initially on pesticide exposure, but currently chronic heat stress and dehydration are considered key etiologic factors. Responding to persistent community and scientific concerns about the role of pesticides, we performed a systematic review of epidemiologic studies that addressed associations between any indicator of pesticide exposure and any outcome measure of CKD. Of the 21 analytical studies we identified, seven were categorized as with low, ten with medium and four with relatively high explanation value. Thirteen (62%) studies reported one or more positive associations, but four had a low explanation value and three presented equivocal results. The main limitations of both positive and negative studies were unspecific and unquantified exposure measurement (‘pesticides’), the cross-sectional nature of most studies, confounding and selection bias. The four studies with stronger designs and better exposure assessment (from Sri Lanka, India and USA) all showed exposure-responses or clear associations, but for different pesticides in each study, and three of these studies were conducted in areas without CKDu epidemics. No study investigated interactions between pesticides and other concommittant exposures in agricultural occupations, in particular heat stress and dehydration. In conclusion, existing studies provide scarce evidence for an association between pesticides and regional CKDu epidemics but, given the poor pesticide exposure assessment in the majority, a role of nephrotoxic agrochemicals cannot be conclusively discarded. Future research should procure assessment of lifetime exposures to relevant specific pesticides and enough power to look into interactions with other major risk factors, in particular heat stress.

**Electronic supplementary material:**

The online version of this article (doi:10.1186/s12940-017-0254-0) contains supplementary material, which is available to authorized users.

## Background

### The global epidemics of chronic kidney disease of unknown etiology (CKDu)

The primary causes of chronic kidney disease (CKD) are diabetes and hypertension, especially in developed countries [1]. However, for more than two decades, various regions of the world have experienced an excess of CKD unrelated to these traditional causes, hereafter referred to as “CKDu” (for CKD of *unknown* cause), in particular in Central America and Mexico (Mesoamerican nephropathy) [2], the North-Central Province of Sri Lanka (Sri Lanka nephropathy) [3] and in the state of Andhra Pradesh of India (Uddanam endemic nephropathy) [4, 5], and possibly in other countries like Egypt [6], Tunisia and Morocco [7], and Saudi Arabia [8].

These regional nephropathies occur mostly in poor adult workers in hot tropical agricultural areas, more frequently among men than women [2, 9]. The most heavily affected populations are sugarcane cutters in Mesoamerica, rice paddy farmers in Sri Lanka, and cashew nut, coconut and rice farmers in India [2, 9]. The nephropathy progresses silently to end-stage renal disease (ESRD) leading to the premature death of thousands of workers [10]. In Central America, national CKD mortality rates in El Salvador and Nicaragua in 2009 were about 12 times higher among men and and eight times higher among women as compared to the USA [11]. In Costa Rica, CKD mortality in the CKDu affected area of Guanacaste was almost five times higher than in the rest of the country during 2008-2012 [12]. Excess mortality is attributed to the CKDu epidemics [11–13]. In Sri Lanka, no mortality statistics have been published specifically for CKD or CKDu. However, in the North and North-Central Provinces of Sri Lanka diseases of the genitourinary system are the leading cause of inhospital deaths (as compared to the 9th cause for the entire country), which is attributed to the CKDu epidemic [14]. Also in India mortality due to the CKDu epidemics is known to be high in the affected areas [15]. From a clinical viewpoint, the regional nephropathies resemble an interstitial tubular pathology, with patients typically being diagnosed in advanced stages of CKD, without diabetes or hypertension, and with no or low-grade proteinuria [5, 16, 17]. The histology has been presented as predominantly interstitial fibrosis and tubular atrophy in studies from El Salvador [18], Sri Lanka [19–21] and India [5]. However, biopsy studies in El Salvador and Nicaragua show important glomerulosclerosis and ischemia with mild to moderate tubulointerstitial damage [22, 23].

Despite clinical, pathological and epidemiologic similarities, as of today it remains uncertain whether the epidemics in different regions of the world correspond to the same disease and whether the causes are the same [9, 24]. In any case, CKDu is now recognized as a serious public health problem to be addressed with renewed efforts in the coming years in Central America [2, 9, 25], Sri Lanka [24] and India [5].

### Pesticides and the search for the etiology of CKDu epidemics

Most researchers believe that the etiology of the unusual CKDu occurrence is multi-factorial [26–29]. In Central America, both occupational and environmental causes have been suggested, including pesticides, heavy metals, nonsteroidal anti-inflammatory drugs (NSAIDs), infections, alcohol, recurrent dehydration due to occupational heat stress, intake of fructose-rich soft drinks, and hyperuricemia and hyperuricosuria [2, 9, 26, 29–33]. The search for the cause of the epidemic was initially focused on pesticides, because CKDu was observed mostly in men in agricultural areas with important pesticide use [30, 34], but the current leading hypothesis is chronic occupational heat stress and dehydration [2, 9, 35]. In Sri Lanka the focus has been almost exclusively on toxic exposures, both heavy metals and pesticides [36–39]. CKDu researchers in Andhra Pradesh, India, have postulated high silica levels in drinking water as a possible cause, either as a consequence of leaching from bedrocks or from pesticides containing silica [15], and recently the combination of silica, strontium and NSAIDs has been proposed [40].

In Central America pesticides have been extensively used for over half a century, yet compliance to regulations is poor [41–43]. Also in Sri Lanka, pesticide use has been high and largely uncontrolled since the green revolution [44], and also in Andhra Pradesh farmers are highly exposed to pesticides [15]. Nonetheless, in Chichigalpa, Nicaragua, where the highest prevalence of CKDu has been documented among men [45], there was no evidence of high levels of any of 57 pesticides analyzed in groundwater, but the study consisted of only one water sample from six locations [46]. A review of toxicological and epidemiologic data for 36 pesticides used historically by the sugarcane company in that specific area did not find a likely agent to explain the epidemic, but the authors indicated that for six pesticides used currently or in the past (2,4-D, paraquat dichloride, captan, cypermethrin, glyphosate and DBCP) there existed strong or good evidence of associations with acute kidney damage [47]. In cane cutters in El Salvador, urinary residues of several relevant pesticides or their metabolites (chlorpyrifos, 2,4-D, pyrethroids) were unremarkable and residues of chlorpyrifos were below the average levels encountered in the Swedish general population for this pesticide (Kristina Jakobsson, University of Gothenburg, personal communication). At the conclusion of the 1st International Mesoamerican Nephropathy (MeN) Workshop in November 2012, pesticides were considered by the participants as an unlikely cause of MeN [2, 48] and, during the last 5 years, recurrent heat stress and dehydration has emerged as a likely key etiologic factor of CKD [2, 9, 29, 35, 48]. However, community concerns about pesticides have persisted and pesticides as a potential cause of MeN continue being subject of debate also among scientists [11, 13, 28, 29, 49–53]. In addition, exposures to toxic agrochemicals (pesticides and fertilizers) remain a leading hypothesis in Sri Lanka [24, 38], and pesticides are considered as a likely cause of excess CKD in Egypt, where outbreaks of CKDu in rural areas have been reported [6]. The first CKDu review published from India recommends to investigate, besides silica and heat stress, also pesticides as a potential etiology [5].

Based on experimental and sometimes clinical evidence, a number of pesticides in common use in many parts of the world are known human nephrotoxins, albeit causes of acute kidney injury (AKI) rather than CKD, in particular glyphosate [54, 55], 2,4-D [56], paraquat [57–59], carbofuran [60], deltamethrin [61], as well as some organophosphates (OP) [62–65] and organochlorine (OC) insecticides [66–68]. Glyphosate has also been shown to trigger epigenetic effects and resulting kidney damage in rats following chronic exposure to ultra-low water concentration of 0.1 ppb of RoundUp [69]. In addition, contamination of commercial formulations of pesticides and fertilizers with heavy metals has been demonstrated in Sri Lanka [37, 44, 70]. Jayasinghe [39] from Sri Lanka went as far as to claim that there is mounting evidence pointing at chemical products used in agriculture, suggesting that CKDu should be renamed “*chronic agrochemical nephropathy”*.

Our aim was to review all available epidemiologic studies that assessed chronic renal effects from agrochemicals to better understand the current evidence for chronic nephrotoxic effects from pesticides in human populations and how such nephrotoxic effects could or could not underlie the *regional epidemics of CKDu* that are appearing globally.

## Approach for evaluating evidence

### Review process

**Fig. 1:**
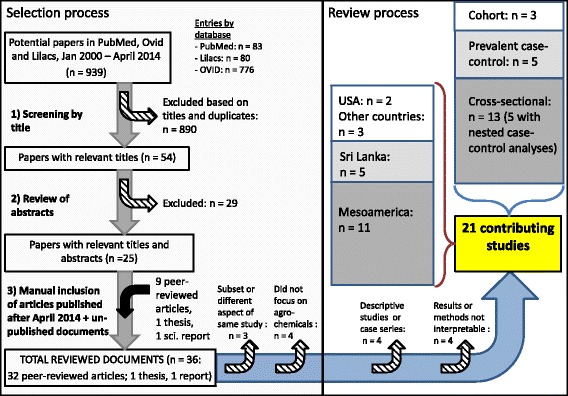
Process of selection and preliminary analysis of relevant studies

We performed a preliminary inspection to define the start of the review process. It appeared that before 2000 studies only referred to general (acute) nephrotoxicity of pesticides and never to CKD or CKDu. Therefore, we conducted a systematic literature review covering the period of January 1st, 2000 to April 30th 2014 (the date the review started) using PubMed, Lilacs, and, through OvidSP, Embase, Medline, Total access collection, EBMR and Global Health databases using a comprehensive list of key terms such as “chronic renal disease”, “agrochemical”, “kidney disease risk factor”, “pesticide”, “fertilizer”, “end-stage renal disease”, “chronic kidney disorder”. The Additional file 1 contain the complete search strategy. Epidemiological studies providing information on the association between occupational or environmental exposure to agrochemicals and the etiology of CKD or ESRD were included, irrespective of what the primary objective of the study was. A first screening identified potentially relevant publications on the basis of their titles. Further analysis of the publications’ abstracts allowed retaining 25 articles. During the review process we kept a scientific watch for the appearance of new publications and, in a second step, this list was manually complemented with 11 other studies published during this time period, nine peer reviewed articles, a thesis and a scientific university report (see Fig. 1). Despite being unpublished, the latter two studies from Nicaragua were included because they were being discussed as evidence among investigators and policy makers in the region. The 36 publications retrieved were organized per chronological order of publication and country or region in which they were conducted, and study characteristics were extracted along with results for associations between pesticide exposures and CKD or CKDu. We also annotated the authors’ conclusions, and commented on the strengths and limitations of the studies. Because the studies were highly heterogeneous and many had important methodological weaknesses, in particular related to exposure assessment, we did not use a scoring system but, based on design and potential bias (regarding pesticides only), we qualitatively concluded on the relative value of the study to contribute to elucidating the role of pesticides in the etiology of CKD or CKDu, as none, low, medium or high. Specific evaluation criteria were strength of study design, adequacy of outcome or case definition, quality of the exposure assessment, clearness and adequacy of statistical analyses; and potential for selection bias, recall bias and confounding. The details of all 36 reviewed documents are in Additional file 2: Table S1.

## Results

### Overview of the reviewed studies

Of the 36 documents, two pairs contained data referring to different aspects of a same study, specifically Orantes-Navarro et al. [71], Orantes et al. [72] and Laws et al. [73, 74]. In addition, Siddharth et al. 2012 [75] was an interim report concerning a subset of a larger population included in an article from 2014 [76]. Of the 33 distinct reviewed studies, three were excluded from further analysis since they did not specifically address agrochemicals (Fig. 1) [77–79].

The remaining 30 studies were analyzed in view of their potential to provide evidence regarding a potential causal relationship between pesticide exposure and the CKDu epidemics or just CKD. Nine were deemed inadequate in this regard and after assignation of an explanation value ‘none’, they were excluded from further analysis. Of these, one was a case series from El Salvador [17] and four were descriptive studies without hypothesis testing, three from El Salvador [80–82] and one from Sri Lanka [37]. In four studies, the results regarding associations of pesticides with kidney disease were not interpretable, either because the methodology underlying the results was not described or because the factual results related to pesticides were not presented: one study each from Mexico [83] and El Salvador [71, 72], and two from Sri Lanka [38, 84] (for details on studies with explanation value ‘none’, see Additional file 2: Table S1). This left 21 studies (23 articles) that analyzed associations between varying pesticide exposures and varying CKD outcomes [6, 19, 36, 45, 73–76, 85–99], 11 from Mesoamerica (Table 1) and 10 from other parts of the world (5 from Sri Lanka, 2 from the USA, and 1 each from Egypt, India and Thailand) (Table 2).

**Table 1:** Mesoamerican studies assessing the role of pesticides in CKD

Reference & country	Study design	Study population	Pesticide exposure assessment	Case definition/outcome(s)	Main findings	Pesticide association ------------- Validity and explanation value^a^
Rugama et al., 2001 [85] Nicaragua	Retrospective hospital-based case-control	CKD hospitalizations during 2000: 165 cases, 334 non-CKD random hospital controls	Pesticide use yes/no, extracted from clinical records	CKD diagnosis at admission	OR pesticide exposure = 5.5 [2.8 – 10.7]	Positive association with pesticide exposure ------------- Prevalent cases; high risk of bias from exposure misclassification; high risk of confounding *Explanation value: low*
Gracía-Trabanino et al., 2005 [86] El Salvador	Cross-sectional survey	Volunteer sample of 353 adult M, 292 coastal and 62 at 500 m above sea level (masl)	Questionnaire: Agricultural occupation yes/no Pesticide exposure yes/no	Proteinuria >15 mg/L CKD defined as SCr ≥1.5 mg/dL among proteinuria positive subjects	For proteinuria: OR agricultural work = 1.62 [0.75-3.49] OR pesticide exposure = 0.79 [0.42-3.47] For SCr ≥1.5: no associations with agricultural work or pesticide exposures	No association with agricultural work No association with pesticide exposure ------------- Cross-sectional; crude pesticide exposure assessment; possible confounding; possible selection bias in second phase *Explanation value: low*
Torres-LaCourt et al., 2008 [88] Nicaragua	Cross-sectional population-based survey	Random sample of 337 adults aged 20-60 (129 M, 208 F) from 2 rural communities	Questionnaire: Current agricultural work yes/no Mixing or applying pesticides yes/no Previous pesticide intoxication yes/no	CKD stage 3 or higher (eGFR <60 ml/min/1.73m^2^)	Results reported separately for the two communities, analyses not adjusted for potential confounders: ORs current agricultural work = 1.87 [0.88-3.99] and 2.68 [1.12-6.39] ORs mixing/applying pesticides = 2.11 [0.99-4.50] and 4.80 [2.33-9.89] ORs previous pesticide intoxication = 1.22 [0.32-4.67] and 1.19 [0.31-4.59]	Positive association with agricultural work Positive association with pesticide exposure No association with previous pesticide intoxication ------------- Cross-sectional; crude exposure assessment; high risk for confounding *Explanation value: low*
Sanoff et al., 2010 [89] Nicaragua	Volunteer screening program with nested case-control analysis	Screening: 997 volunteers aged >18 y (848 M, 149 F) Case-control: 334 M, 112 cases	Questionnaire: Field labor yes/no Work with or exposure to pesticides yes/no	eGFR Screening: <60 *vs* ≥ 60 ml/min/1.73m^2^ Case control: <60 *vs* ≥ 80 ml/min/1.73m^2^	Screening: OR agricultural field labor = 2.48 [1.59-3.89] OR pesticides = 1.38 [0.90-2.11] Case -control: OR agricultural field labor = 2.38 [1.44-3.93] OR pesticides = 1.57 [0.97-2.55]	Positive association for agricultural field labor Weak positive association with pesticide exposure ------------- Screening survey; crude exposure assessment; insufficient adjustment for potential confounders; likely selection bias *Explanation value: medium*
O’Donnell et al., 2011 [90] Nicaragua	Cross-sectional population-based survey; nested case-control analysis	Random sample of 771 individuals aged ≥18 (298 M, 473 F) from 300 eligible households Case-control: 98 cases, 221 controls	Questionnaire: Agricultural work yes/no Pesticide exposure yes/no Mixing or applying pesticides yes/no	CKD ≥ stage 3 (eGFR <60 ml/min/1.73m^2^)	Unadjusted/sex and age adjusted logistic regressions: OR agricultural work = 2.09 [1.08-4.05]/1.00 [0.44-2.27]) OR any pesticide exposure = 2.45 [1.31 – 4.57]/1.85 [0.84, 4.07]) OR mixing or applying pesticides = 1.78 [1.09 – 2.91]/1.32 [0.66-2.64]	No association with agricultural work Weak positive association with any pesticide exposure No association with mixing or applying pesticides ------------- Cross-sectional; crude exposure assessment; likely confounding; likely selection bias *Explanation value: medium*
Orantes et al., 2011 [91] El Salvador	Community screening and cross-sectional survey	775 individuals age ≥ 18, (343 M, 432 F)	Questionnaire: Agricultural occupation yes/no Contact with agrichemicals yes/no	CKD stages 1-5 (2 determinations with a 3-month interval)	OR agricultural occupation = 1.35 [0.63–2.88] OR contact with agrichemicals = 1.23 [0.66 – 2.31]	No association for agricultural occupation No association for contact with agrichemicals ------------- Cross-sectional; crude exposure assessment; risk for confounding; incomplete strategy for statistical analyses *Explanation value: medium*
Laux et al., 2012 [93] Nicaragua	Community-based cross-sectional survey	267 adults (120 M, 147 F)	Questionnaire: Work with pesticides yes/no	Proteinuria	OR work with pesticides = 1.09 [0.6–1.98]	No association with pesticide exposure ------------- Cross-sectional; crude exposure assessment; study conducted in non-CKDu area *Explanation value: medium*
Raines et al., 2014 [45] Nicaragua	Cross-sectional population-based survey; nested case-control analysis	424 adults (166 M, 258 F) 280 in case-control analysis (78 cases)	Questionnaire: Agricultural worker yes/no Among subset of agricultural workers: Lifetime days of: -mixing pesticides -applying pesticides Self-reported history of accidentally inhaling pesticides Degree of use of personal protective equipment (PPE)	eGFR <60 ml/min/1.73 m^2^ Case-control: <60 vs ≥ 90 ml/min/1.73 m^2^	OR agricultural worker 2.05 [0.61-6.90] Subset agricultural workers: Lifetime days of mixing and applying pesticides: *p* = 0.13 and *p* = 0.22 respectively Level of PPE: *p* = 0.35 OR for accidental inhalation of pesticides = 3.14 [1.12 – 8.78]	Weak association with agricultural work No association with main pesticide exposure indicators No association with PPE Accidental inhalation of pesticides associated with low eGFR, but pesticide inhalation without further specifications is not interpretable ------------- Cross-sectional; unclear exposure indicators, possible confounding *Explanation value: medium*
Laws et al., 2015 & Laws et al., 2016 [73, 74] Nicaragua	Cohort	284 sugarcane workers (251 M, 33 F), incl. 29 agrichemical applicators	Job title: agrichemical applicator	eGFR (ml/min/1.73m^2^) Biomarkers of early kidney injury NGAL, NAG, IL-18, albuminuria	Mean change eGFR for pesticide applicators during harvest season −3.8 (−9.9, 2.3) Mean changes of early injury markers for pesticide applicators during harvest season: NGAL −0.1 μg/g (*p* = 0.9) NAG -0.12 μg/g (*p* = 0.6) IL-18 -1.2 ng/g (*p* = 0.6) ACR +0.3 mg/g (*p* = 0.8)	No association between a job of spraying pesticides with decrease in kidney function or increase in indicators of early tubular damage over one cutting season ------------- Cohort design with short 6-month follow up; crude assessment with jobtitle for current exposure *Explanation value: medium*
García-Trabanino et al., 2015 [94] El Salvador	Cross-sectional occupational survey	189 sugarcane cutters (168 M, 21 F)	Questionnaire: Pesticide use yes/no Use of specific pesticides yes/no: Herbicides: glyphosate, paraquat, 2,4-D, triazines Insecticides: specific organophosphates, carbamates, pyrethroids	eGFR <60 ml/min/1.73 m^2^	Ever use of any pesticide not associated with low eGFR Ever use of carbamate insecticides: 74% among workers with reduced eGFR vs 29% among remaining workers and a significant predictor in multivariate model	Association with ever use of carbamate insecticides No association with other groups of pesticides ------------- Cross-sectional; exposure assessment specific for chemical groups, but unquantified; multiple comparisons *Explanation value: medium*
Wesseling et al., 2016 [99] Nicaragua	Occupational cross-sectional study	86 sugarcane cutters, 56 construction workers, 52 subsistence farmers, all males	Questionnaire: Pesticide use ever yes/no Use of specific pesticides yes/no: glyphosate, paraquat, 2,4-D, chlorpyrifos, cypermethrin	eGFR <80 ml/min/1.73 m^2^	Ever use of any pesticide and ever use of specific pesticides not associated with reduced eGFR, for all workers combined and in analyses restricted to cane cutters	No association with ever use of pesticides No association with any of the specific pesticides ------------- Cross-sectional; exposure assessment specific for chemical groups, but unquantified *Explanation value: medium*

*Abbreviations*: *CKD* chronic kidney disease (u: of unknown etiology; nt: not related to traditional risk factors), *eGFR* estimated glomerular filtration rate, *ESRD* end-stage renal disease, *F* female, *M* male, *OR* odds ratio [95% confidence interval], *SCr* serum creatinine, *NGAL* neutrophil gelatinase-associated lipocalin, *NAG* N-acetyl-D-glucosaminidase, *IL-18* interleukin-18, *ACR* urinary albumin-to-creatinine ratio

^a^Explanation value: The study’s ability to contribute to knowledge about potential associations between pesticides and CKD or CKDu (according to the objective of the study), based on a qualitative evaluation of design and the validity of the results. For details see Additional file 2: Table S1 and the main text

**Table 2:** Studies from Sri Lanka and other non-Mesoamerican countries assessing the role of pesticides in CKD

Reference & country	Study design	Population	Exposure assessment	Case definition/outcome(s)	Main findings	Pesticide association ------------- Validity and explanation value^a^
Sri Lanka						
Peiris-John et al., 2006 [87] Sri Lanka	Cross-sectional	4 groups: 23 OP-exposed farmers with chronic renal failure (CRF) vs 18 unexposed patients with CRF vs 239 OP-exposed farmers without CRF vs 50 unexposed fishermen without CRF	Red blood cell acetyl cholinesterase (AChE) levels (U/g) as proxy of organophosphate exposures	CRF (not further specified)	Significant differences in AChE levels: exposed CRF (18.6 U/g) < unexposed CRF (26.6) < exposed non-CRF (29.1) < non-exposed non-CRF (32.6)	Possible association between long-term low-level OP-exposures, cholinesterase levels and CKD -------------- Exploratory aim with unconventional cross-sectional design; inadequate selection of study participants; high risk of bias from exposure misclassification; high risk of confounding *Explanation value: low*
Wanigasuriya et al., 2007 [36] Sri Lanka	Hospital- based case – control (prevalent cases)	183 CKDu cases (136 M, 47 F), 200 controls among HT and DM patients (139 M, 61 F), age 36-67	Questionnaire: Farmer yes/no Pesticide exposure yes/no Drinking water source (well-water home, well-water field, pipe born)	SCr > 2 mg/dL	Bivariate analyses: Males: OR farmer = 4.68 [2.50- 8.82) OR pesticides = 2.94 [1.73-5.01] OR drinking well-water field = 1.72 [0.92-3.22] Females: OR farmer = 1.28 [0.55-2.99) OR pesticides: 0 cases OR drinking well-water home = 4.24 [1.51-12.32] Multivariate logistic regressions: NO associations for farming, pesticide use and drinking well-water	No associations in multivariate analyses with farming, pesticide use and well-water ------------- Prevalent cases; high risk of bias from exposure misclassification; inadequate reporting of statistical analyses and pesticide results *Explanation value: low*
Athuraliya et al., 2011 [19] Sri Lanka	Cross-sectional population-based survey with case –control analyses	6153 (2889 M, 3264 F): age >19 ᅟ CKDu endemic area Medawachchiya 2600 Two non-endemica areas Yatinuwara708 Hambantota 2844 ᅟ 109 CKDu patients in Medawachchiya (66 M, 43 F)	Questionnaire -Farmer yes/no -Spraying or handling agrochemicals yes/no	Proteinuric chronic kidney disease	Entire study population: Adj OR farmer 2.6 (1.9–3.4) Adj OR agrochemical exposure 2.3 (1.4–3.9) Medawachchiya (CKDu region) Adj OR farmer 2.1 (1.4–3.3) Adj OR agrochemical exposure 1.1 (0.7–1.9) ᅟ Yatinuwara (non-CKDu region) Adj OR farmer 1.5 (0.5–3.9) Adj OR agrochemical exposure 1.6 (0.8–3.2) ᅟ Hambantota (non-CKDu region) Adj OR farmer 1.6 (1.0–2.7) Adj OR agrochemical exposure 5.6 (2.3–13.2)	Pesticide use was not associated to proteinuric CKD in the CKDu region, but it was associated to CKD of known causes in one of the two non-CKDu regions. ------------- Cross-sectional, crude pesticide exposure assessment, misclassification of disease *Explanation value: medium*
Wanigasuriya et al., 2011 [92] Sri Lanka	Cross-sectional population-based survey with case –control analyses	886 (461 M, 425 F) household members aged ≥18	Questionnaire: Farmer yes/no Pesticide spraying yes/no Drinking water source	Micro-proteinuria	Bivariate analyses: OR farmer = 1.38 [0.71- 2.70) OR pesticides = 1.01 [0.60-1.72] OR well-water in the field = 1.79 [1.07-3.01] ᅟ Multivariate logistic regression: OR pesticides = 0.43 [0.21-0.90] OR well-water in the field = 1.92 [1.04-3.53]	Positive association with drinking from well-water in the field Negative association with pesticide spraying ------------- Cross-sectional, crude pesticide exposure assessment, misclassification of disease Explanation value: medium
Jayasumana et al., 2015 [95] Sri Lanka	Hospital-based case-control (prevalent cases)	125 cases (89 M, 36 F), 180 controls (98 M, 82 F)	Questionnaire: Usual occupation last 10 years, farming yes/no Use of fertilizer and specific pesticides over last 10 years yes/no (organophosphates, paraquat, MCPA, glyphosate, bispyribac, carbofuran, mancozeb and other common pesticides) Glyphosate, metals and hardness measured in water of serving and abandoned wells	CKDu	Bivariate logistic regression with significantly increased ORs for farming, use of fertilizers, and use of organophosphates, paraquat, MCPA, glyphosate, bispyribac and mancozeb ᅟ Multivariate logistic regression: OR drinking well water = 2.52 [1.12-5.70] OR history drinking water from abandoned well = 5.43 [2.88-10.26] OR pesticide application = 2.34 [0.97- 5.57] OR use of glyphosate = 5.12 [2.33-11.26] ᅟ Water hardness: abandoned wells: very high; serving wells: moderate to hard; reservoir and pipeline: soft Glyphosate concentration in water from abandoned well significantly higher than in serving wells (median 3.2 μg/L and 0.6 μg/L, respectively).	Positive association with pesticide applications Positive association with use of glyphosate Positive association with drinking well-water and, especially, with history of drinking water from abandoned (with hardest water and highest glyphosate levels) -------------- Prevalent cases; relatively good case ascertainment; specific, unquantified pesticide exposure assessment Exposure response for glyphosate in water; control of potential confounders *Explanation value: high*
Other countries						
Kamel & El-Minshawy, 2010 [6] Egypt	Hospital-based case-control (prevalent cases)	216 ESRD cases (141 M, 75 F) from unknown cause 220 random controls (152 M, 68 F) from other patients	Questionnaire: Rural residency yes/no Drinking unsafe (non-pipe) water yes/no Farming occupation yes/no Pesticide exposures by any mean yes/no	ESRD of unknown cause (clinical exams)	Bivariate analyses: rural living, drinking unsafe water, being a farmer and pesticide exposure associated with ESRD (*p* < 0.001) Multivariate analyses (model not specified): OR pesticide exposure 2.08 [1.42 – 3.06]	Possible association with pesticide exposures ------------- Prevalent cases; no data to evaluate potential selection bias; crude exposure assessment, statistical methods not well described *Explanation value: low*
Siddharth et al., 2012 [75] India Note: this study is an interim report of Siddarth *et al.*, 2014 [76]	Hospital-based case-control (prevalent cases)	150 CKD cases (77 M, 73 F): patients attending nephrology departments 96 controls (51 M, 45 F): staff or persons accompanying CKD patients in the hospital Age 30-50	Levels of organochlorine (OC) pesticides in blood	CKDu: eGFr <60 ml/min/1.73m^2^ for >3 months Oxidative stress markers	Significantly higher blood levels in cases for α–HCH, γ-HCH, total HCH, α-endosulfan, β-endosulfan, aldrin, p,p’-DDE, and TPL. Among cases, adjusted Spearman correlations between eGFR and different pesticide analytes varied between −0.07 and −0.23 (significant for γ-HCH, total HCH and aldrin). When adjusting additionally for levels of other analytes, the association with eGFR remained significant only for aldrin. In addition, significant correlation between eGFR and TPL (*r* = −0.26).	Association of blood levels of OCs (from environmental exposures) with CKDu, mediated partially through genotype -------------- Prevalent cases; specific and quantitative assessment for non-occupational exposures to OCs, study in a non-CKDu setting; some potential for inverse causation; low risk for confounding *Explanation value: high*
Siddarth et al., 2014 [76] India	Hospital-based case-control (prevalent cases)	270 cases (140 M, 130 F): patients attending nephrology departments 270 age and sex matched controls: staff or persons accompanying CKD patients in the hospital	Concentrations of organochlorine pesticides in blood GST genotyping	CKDu: eGFR <90 ml/min/1.73m^2^ with or without proteinuria, for 3 months	Cases had significantly higher blood concentrations of α–HCH, γ-HCH, total HCH, α-endosulfan, β-endosulfan, aldrin, p,p’-DDE, and total pesticides Significant associations with CKDu for 3rd versus 1st tertile for α-HCH (OR = 2.52), γ-HCH (OR = 2.70), total-HCH (OR = 3.18), aldrin (OR = 3.07), α-endosulfan (OR = 2.99), and β-endosulfan (OR = 3.06). Total pesticides 3rd to 1st tertile OR = 2.73 [(1.46–9.47). CKDu patients having either one null or two null genotypes tend to accumulate majority of pesticides, whereas in healthy controls only in the subset with both null genotypes for some pesticides.	
Lebov et al., 2016 [97] USA	Cohort (follow-up since 1993-1997)	55,580 licensed pesticide applicators (320 ESRD)	Self-administered questionnaires: Ordinal categories of intensity-weighted lifetime days for 39 specific pesticides Pesticide exposure resulting in medical visit or hospitalization Diagnosed pesticide poisoning High level pesticide exposure event	ESRD	Significantly increased HR for highest category of use vs non-users and significant exposure-response trends: Alachlor HR = 1.51 [1.08-2.13], p for trend 0.015 Atrazine HR = 1.52 [1.11-2.09], p for trend 0.008 Metolachlor HR = 1.53 [1.08-2.13], p for trend 0.008 Paraquat HR = 2.15 [1.11-4.15], p for trend 0.016 Pendimethalin HR = 2.13 [1.20-3.78], p for trend 0.006 Permethrin HR = 2.00 [1.08-3.68], p for trend 0.031 More than one medical visit due to pesticide use HR = 2.13 [1.17 - 3.89], p for trend for increasing number of doctor visits 0.04. Hospitalization due to pesticide use HR = 3.05 [1.67 to 5.58]	Association between use of specific pesticides and ESRD Association between ESRD and exposures resulting in medical visits or hospitalization and ESRD -------------- Large cohort with long follow-up; study in non-CKDu endemic regions; specific and quantitative exposure assessment; multiple comparisons; low risk for confounding *Explanation value: high*
Lebov et al., 2015 [96] USA	Cohort (follow-up since 1993-1997)	31,142 wives of licensed pesticide applicators (98 ESRD)	Self-administered questionnaires or telephone interview -direct exposures (*n* = 17,425): ordinal categories of intensity weighted lifetime use of any pesticide, 10 specific pesticides and 6 chemical classes -Indirect pesticide exposures (husband’s pesticide use) among wives without personal use (*n* = 13,717) -Indicators of residential pesticide exposure	ERSD	Highest category of cumulative lifetime-days of pesticide use in general vs never personal use: HR 4.22 [1.26-14.2] Exposure-response trends for husband’s use of paraquat HR 1.99 [1.14-3.47] and butylate HR 1.71 [1.00-2.95] No excess risk for indicators of residential exposures	Association between direct general pesticide use and husband’s use of paraquat and ESRD in women No associations with residential exposures --------------- Large cohort with long follow-up; study in non-CKDu endemic regions; specific and quantitative exposure assessment; multiple comparisons; low risk for confounding *Explanation value: high*
Aroonvilairat et al., 2015 [98] Thailand	Cross-sectional	64 workers of orchids (30 M, 34 F) and 60 controls (33 M, 27 F)	Mixing and spraying pesticides during work at orchard for at least three months	Difference in BUN and SCr	BUN (mg/dL) exposed 12.64 ± 3.7 (3.7% abnormal) vs BUN unexposed 12.43 ± 2.9 (1.7% abnormal), *p* = 0.76 SCr (mg/dL) exposed females 0.86 ± 0.11 (3.7% abnormal) vs unexposed females 0.82 ± 0.11 (2.9% abnormal), *p* = 0.11 SCr exposed males 1.09 ± 0.11 (0% abnormal) vs unexposed males 1.09 ± 0.10 (0% abnormal), *p* = 0.95	No association between occupation in highly pesticide exposed farming and decreased kidney function ------------- Cross-sectional; crude exposure assessment, selection of study population not well described; no confounding adjustment *Explanation value: low*

*Abbreviations*: *AChE* red blood cell acetylcholinesterase, *ACR* albumin to creatinine ratio, *ANOVA* analysis of variance, *CKD* chronic kidney disease (u, of unknown etiology), *BUN* blood urea nitrogen, *CRF* chronic renal failure, *DB* diabetes, *DW* drinking water, *DDE* dichlorodiphenyldichloroethylene, *eGFR* glomerular filtration rate, *ESRD* end-stage renal disease, *F* female, *GST* glutathione-S-transferase, *HCH* hexachlorocyclohexane, *HT* hypertension, *M* male, *MVLR* multivariate logistic regression, *OP* organophosphate pesticides, *SCr* serum creatinine

^**a**^Explanation value: The study’s ability to address potential associations between pesticdes and CKD or CKDu. For details see Additional file 2: Table S1 and the main text

### Methodological aspects of the reviewed studies

Tables 1 and 2 summarize basic epidemiologic characteristics of the 21 studies. With regard to *study design*, 13 studies were cross-sectional in nature [19, 45, 86–94, 98, 99], including five studies, − four population-based surveys [19, 45, 90, 92] and a screening program [89] -, that also performed nested case-control analyses. Five studies had a case-control design, all hospital-based and with prevalent cases [6, 36, 75, 76, 85, 95]. Only three studies had a longitudinal design, a prospective cohort during one harvest season among Nicaraguan sugarcane workers [73, 74] and two prospective cohorts of the USA Agricultural Health Study (AHS) among licensed pesticide applicators in Iowa and North Carolina and their wives, respectively, with a follow-up of more than 15 years [96, 97].

Depending on the design, the *study populations* comprised entire communities or a subset, volunteers, or groups of farmers or agricultural workers, in Mesoamerica especially sugarcane workers. Cases were often hospital CKD or CKDu patients, and controls most often patients with other diagnoses. The studies under scrutiny used many different *markers and definitions of CKD*, most often proteins in urine, serum creatinine (SCr) and CKD stages based on estimated glomerular filtration rate (eGFR), and a single study also early markers of tubular injury. All studies in Sri Lanka, India and Egypt were restricted to CKDu cases whereas in Mesoamerica and other countries, all cases of CKD were included in the studies independently of their cause.

With regard to *exposure assessment*, practically all studies focused on pesticide exposures in occupational settings and in the majority the exposure assessment was extremely crude. Eleven studies only had a dichotomous yes/no exposure variable of pesticide use without any specification of pesticidal agents or any quantification of duration and/or intensity of exposure over the lifetime [6, 19, 36, 85–87, 89, 91–93, 98]. Several studies used a proxy of high exposures, specifically the job title of pesticide applicator [73, 74, 96, 97], a history of self-reported pesticide poisoning [45, 88, 97], and an index of life-time days of mixing-spraying pesticides without specification of pesticidal agents [45, 96, 97], whereas three studies assessed the effects of a number of specific pesticidal agents but without quantification of their use [94, 95, 99]. One of these latter studies, a case-control from Sri Lanka, combined questionnaire data about source of drinking water with levels of glyphosate residues and hardness of the water to evaluate a gradient of exposure levels [95]. Only the two cohorts of the AHS in the USA computed intensity weighted lifetime use for specific pesticidal agents or groups of chemicals, defined as the product of frequency and duration of use, modified by an intensity factor to account for differences in application practices [96, 97]. A single study used biomarkers, i.e. blood concentrations of OC pesticides or their metabolites [75, 76], focusing on non-occupational exposures in Delhi, India. Lebov et al. [96] also examined several indicators of non-occupational exposures among wives of licensed applicators in the USA.

Many of the reviewed studies had no or inadequate control of potential confounding; selection bias related to volunteer participation, high non-participation, or the use of inadequate case or control groups; possible recall bias; and deficient description of statistical analyses (see Additional file 2: Table S1). We classified the explanation value of seven of the studies as relatively low, ten as medium, and four as relatively high (Table 3).

**Table 3:** Reviewed studies ranked by their explanatory potential on the etiological role of pesticide for CKD/CKDu

Study	CKD marker	Potential to explain pesticide role in CKD/CKDu			Associations
		Low	Medium	High	
		Pesticide exposure indicator			
Rugama, 2001 [85]	CKD diagnosis at hospital admission	Pesticide use			Positive
Gracía-Trabanino et al., 2005 [86]	Proteinuria >15 mg/L	Pesticide use			No
	SCr >1.5 mg/dL	Pesticide use			No
Peiris-John et al., 2006 [87]	Chronic renal failure diagnosis at hospital	Acetyl cholinesterase levels in four groups (exposed CRF, unexposed CRF, exposed non-CRF and unexposed non-CRF)			Positive
Wanigasuriya et al., 2007 [36]	CKDu hospital diagnosis	Pesticides			No
Torres-Lacourt et al. 2008 [88]	eGFR <60 ml/min1.73/m^2^	Pesticide use			Positive
		Pesticide intoxication			No
Kamel & El Minshawy, 2010 [6]	ESRDu	Pesticide exposure			Positive
Aroonvilairat et al., 2015 [98]	BUN and SCr	Pesticide mixing and spraying in orchid for at least three months			No
Orantes et al., 2011 [91]	Persistent CKD stages 1-5 determined twice with 3-months interval		Contact with agrichemicals		No
Wanigasuriya et al., 2011 [92]	Micro-proteinuria		Pesticides		No
Laux et al., 2012 [93]	Proteinuria		Work with pesticides		No
Laws et al., 2015 & 2016 [73, 74]	Change in eGFR (ml/min/1.73 m2)		Job as pesticide applicator over 6-month period		No
	Change in early kidney injury markers				No
Wesseling et al., 2016 [99]	eGFR <80 ml/min/1.73m^2^		Any pesticide use		No
			Specific pesticides: glyphosate, paraquat, 2,4-D, chlorpyrifos, cypermethrin		No
Sanoff et al., 2010 [89]	eGFR <60 ml/min/1.73m^2^		Pesticides		Weak positive
O’Donnell et al., 2011 [90]	eGFR <60 ml/min/1.73m^2^		Any pesticide exposure		Weak positive
			Mixing/applying pesticides		No
Athuraliya et al., 2011 [19] Sri Lanka	Proteinuric CKD		Pesticides		Negative in CKDu endemic area Positive in non-endemic area
Raines et al., 2014 [45]	eGFR <60 ml/min/1.73m^2^		Lifetime days mixing/applying		No
			History of accidentally inhaling pesticides		Reported positive, but not interpretable
García-Trabanino et al., 2015 [94]	eGFR <60 ml/min/1.73m^2^		Any pesticide use		No
			Carbamate insecticides		Positive
			Glyphosate, paraquat, 2,4-D, triazines, organo-phosphates, pyrethroids		No
Jayasumana et al., 2015 [95]				Use of fertilizers, organo-phosphates, paraquat, MCPA, bispyribac, mancozeb	Positive only in unadjusted analyses
				Use of glyphosate	Positive also in multivariate analyses
				Drinking water from serving wells and from abandoned wells (hardest water and highest glyphosate levels)	Positive with dose response
Siddharth et al., 2012 and Siddharth et al., 2014 [75, 76]	CKDu with eGFR <60 ml/min/1.73m^2^ for >3 months			Urinary organochlorine pesticides and metabolites and interaction with GST polymorphism	Positive
Lebov et al., 2016 [97]	ESRD among male applicators			Intensity weighted lifetime days for 39 pesticides: Alachlor, atrazine, metalochlor, paraquat, pendimethalin, permethrin	Positive with dose-response
				Petroleum oil, imazethapyr, coumaphos, parathion, phorate, aldicarb, chlordane, and metalaxyl	Weak positive without dose responses
				Glyphosate and 24 other pesticides	No
				Pesticide exposure resulting in medical visit or hospitalization	Positive
				Diagnosed pesticide poisoning	No
				High level pesticide exposure event	No
Lebov et al., 2015 [96]	ESRD among wives of licensed applicators			Intensity weighted lifetime days for applying -Pesticides in general	Positive
				-Specific pesticides	No
				Husband’s use of paraquat	Positive
				Residential exposure	No

### Findings and validity of the reviewed studies

Thirteen studies (62%) reported one or more positive associations between a pesticide exposure indicator and an indicator of CKD: four studies with a relatively low, five with a medium, and all four with a relatively high explanation value. Of the eight negative studies, three had a low explanation value and five were considered to have a medium explanation value (Table 3).

#### Studies considered with relatively low explanation value

Regarding the four studies with lower explanation value that reported a positive association between pesticides and CKD [6, 85, 87, 88], control of potential confounding was absent in three and inadequate in one study (no adjustment for age despite controls being 10 years older), and in three of these studies the exposure assessment was based on one single dichotomous ‘pesticide’ exposure variable. One could argue that the studies with ‘pesticide’ as the exposure variable could have given rise to a bias of non-differential exposure misclassification and that the true risk was higher than the observed. However, without control of potential confounding, an alternative explanation could also be that ‘pesticides’ correlate with other agricultural exposures, in particular heat stress. The fourth study [87] compared red blood cell acetylcholinesterase (AChE) levels among four groups, with and without OP exposure and with and without chronic renal failure (CRF). An important limitation of this study was that the unexposed groups were participants of other studies in other regions. The three negative studies with low explanation value had, besides non-specific and non-quantified exposure assessment, multiple other sources of potential bias (see Additional file 2: Table S1). The negative study from Thailand compared a group of farm workers highly exposed to pesticides on a daily basis (not all, 88%) with an undefined group of non-farmers from the same region without consideration of potential confounding [98]. The negative studies from El Salvador and Sri Lanka used proteinuria as a marker for CKDu, although CKDu is basically a non-proteinuric disease, leading to incomplete case detection and possible selection bias [86, 92].

#### Studies considered with medium explanation value

Of the 10 studies with a medium explanation value, five did not observe any association and five reported some positive association, albeit with equivocal or ambiguous results in three studies. All studies in this category had a cross-sectional design, except one negative cohort in Nicaragua.

Of the studies with a positive finding, three were community-based surveys conducted in the municipalities of León and Chinandega in Nicaragua [45, 89, 90], the region with the highest CKDu occurrence of Mesoamerica, especially among men [45]; one was an occupational cross-sectional survey among cane cutters in a CKDu epidemic area in El Salvador [94] and one a population-based survey in CKDu endemic and non-CKDu regions in Sri Lanka [19]. One of the studies in the Nicaraguan hotspot observed a weak association between ‘pesticide’ exposure both in data obtained through screening of volunteers (odds ratio (OR) =1.4, 95% confidence interval (CI) 0.9-2.1) and in a nested case-control analysis restricted to male participants (OR 1.6, 95% CI 1.0-2.6) [89]. This study controlled for confounding factors, and besides its crude exposure assessment, its main limitation was that study participants were volunteers and the authors did not address how a possible selection bias could have affected their results. The study in the hotspot in El Salvador found that ‘ever use of carbamate insecticides’ was more common among cane cutters with reduced eGFR than among cutters with normal eGFR (74% vs 29%) and carbamate use was a significant predictor for reduced eGFR in multivariate analyses [94]. This study was negative for all other specific pesticides or groups of pesticides that were examined qualitatively, including the herbicides glyphosate and paraquat. With regard to the three studies with equivocal or ambiguous results [19, 45, 90], in Nicaragua a non-significant increased risk of CKD stage ≥3 (OR 1.9, 95% CI 0.8-4.1) was found for ‘any pesticide exposure’, whereas no association was found for ‘applying and mixing pesticides’, the latter indicator likely reflecting higher exposures than the former [90]. In the hotspot of Chinandega, Raines et al. [45] reported a significant association of reduced kidney function with a vague exposure indicator ‘ever accidentally inhaling pesticides’ (OR 3.3, 95% 1.3-8.3) among agricultural workers, but did not find a relationship of CKD with a semi-quantitative exposure measure of life-time days of pesticide applications. The fifth study with a positive result, from Sri Lanka, reported an association between non specific and unquantified pesticide use and proteinuric CKD after adjusting for confounding (OR 2.3, 95% CI 1.4 – 3.9) but when stratifying by region the association was restricted to Hambantota, an area with low prevalence of CKDu (OR 5.6, 95% CI 2.3 – 13.2), whereas no association was observed in Medawachchiya, an area in the North Central province with high prevalence of CKDu (OR 1.1, 95% CI 0.7-1.9) [19].

Concerning the five negative studies with medium explanation value, their main limitation was the crude exposure assessment, three with a dichotomous pesticide exposure variable [91–93], one with jobtitle of pesticide applicator indicative only of current exposure [73, 74], and one with unquantified exposure of specific pesticides [99]. One negative study from Sri Lanka used proteinuria as a marker for CKD although CKDu is basically a non-proteinuric disease, possibly causing selection bias [86, 92]. One Nicaraguan study was conducted in a high-altitude non-CKDu area, not finding CKDu cases [93]. Another negative Nicaraguan study, a cohort of sugarcane workers, was conducted in the same hotspot of MeN as studies mentioned above [73, 74]. It compared change in SCr or eGFR and in markers of early tubular injury over the course of a 6-month harvest season beween workers performing different tasks. Pesticide applicators did not present any changes, in contrast with cutters and seeders exposed to extreme heat, whose eGFR did significantly decrease together with an increase of markers of early kidney damage. However, the limitation of using job title for current exposure without further specifications of exposure and its modifying factors was not addressed.

#### Studies considered with relatively high explanation value

The four studies (five articles) with a relatively high explanation value all reported a positive association between one or more pesticide exposure indicators and different markers of CKD [75, 76, 95–97]. One case-control study in a CKDu endemic area in Sri Lanka found a significant association with overall pesticide application (OR 2.3, 95% CI 1.0-5.6) and use of glyphosate (OR 5.1, 95% CI 2.3-11.3), adjusted for age, sex, education, family CKD and exposure modifiers [95]. Although this study did not quantify the use of specific pesticides, it was the only one among all those conducted in CKDu endemic areas that investigated a potential exposure-response relationship by combining questions on water intake from different sources in relation to water hardness and levels of the herbicide glyphosate detected in water. With drinking pipe water or reservoir water with soft water and with traces or no detection of glyphosate as the reference, drinking from serving wells with hard water and intermediate concentrations of glyphosate (median 0.6 μg/L) yielded an adjusted OR of 2.5 (95% CI 1.1-5.7), and drinking from abandoned wells with very hard water and highest concentrations of glyphosate (median 3.2 μg/L) yielded an adjusted OR of 5.5 (95% CI 2.9-10.3).

The three remaining studies with a higher explanation value were conducted in non-CKDu regions. The study in Delhi, India, found an association between blood concentrations of OCs and their metabolites with CKDu, in particular for alpha- and gamma-hexachlorocyclohexane, aldrin, and alpha- and beta-endosulfan [75, 76]. This study was conducted in an urban setting and, in addition, all subjects occupationally exposed to pesticides were excluded. Therefore, the observed relationships between OCs and CKDu can only derive from dietary and environmental exposures. The cohort of the AHS among licensed applicators in the USA observed significant exposure-response relationships of ESRD with all pesticides combined and specific pesticides (alachlor, atrazine, metalochlor, paraquat, pendimethalin, and permethrin) as well as increased risks in relation to more than one medical visit and hospitalization due to any pesticide use [97]. The cohort among the wives of licensed applicators showed an exposure-response relationship between ESRD and all pesticides combined among the subset of women who ever sprayed pesticides, and a significant exposure-response association between ESRD and the husband’s cumulative uses of paraquat and butylate among the women who had never worked with pesticides [96].

## Discussion

### The epidemiologic evidence for pesticides as a causal factor in CKDu

In this review we included to the best of our knowledge all epidemiologic studies conducted this century that examined an association between pesticide exposures and any of a variety of outcomes indicating CKD. However, few of the 21 analytical studies had a robust design and, although 13 (62%) of the studies reported one or more positive associations, 4 of these studies were of low quality [6, 85, 87, 88], 3 had equivocal results [19, 45, 90] and 3 were not conducted in a CKDu-endemic region [75, 76, 96, 97]. In general, the heterogeneity in study designs, exposure assessment and outcomes or case definitions, together with important bias in most studies, severely limit the interpretation of both positive and negative results and the comparisons between these studies. In particular the unquantified and/or unspecific pesticide exposure assessment contributed to low quality of the studies. ‘Pesticides’ is a questionable exposure measure, because pesticides are a group composed of hundreds of toxins with distinct toxicological actions. In addition, the use of pesticidal agents varies enormously between crops, regions and over time, as well as exposure determinants such as mixing and application methods, use of personal protective equipment, and storage and disposal practices. None of the studies conducted in CKDu endemic or epidemic areas examined all such factors in depth and most of these studies, either positive or negative, add only marginally to the evidence in pro or con of a causal association between pesticides and CKD or CKDu, due to their methodological limitations. Even if toxicological data demonstrate the nephrotoxicity of specific pesticides, deficient exposure assessment may result in false negative studies [100].

Positive associations between pesticides and CKD or CKDu became more apparent with stronger designs and better exposure assessments but, of the four studies classified as with higher explanation value, three were conducted in non-CKDu regions limiting the generalization of these results to areas with high prevalence of CKDu. Of the three remaining positive studies, one reported in Nicaragua a weak relationship between eGFR <60 and any nonspecific and unquantified pesticide exposure [90], one reported in El Salvador an association of reduced eGFR with carbamate insecticides [94], and the strongest study by Jayasumana et al. [95] implicated glyphosate as a causal agent in the CKDu epidemic in Sri Lanka.

Conversely, none of the negative studies was classified as with a higher explanation value. The strongest evidence against a pesticide association with CKDu epidemics has been provided by the cohort of Nicaraguan sugarcane workers, which did not show kidney effects during the 6-month follow-up of pesticide applicators [73, 74]. Whether or not such a short period of exposure could have triggered kidney damage was not discussed, and the study did not include individual exposure measures. Two cross-sectional studies, conducted in the same area, did not observe associations between reduced eGFR and days of pesticide use over a lifetime [45] and use of several specific pesticides including glyphosate and paraquat [99], respectively. However, not one of the negative studies had a comprehensive exposure assessment.

When taking a closer look at the four studies classified as with higher explanation value, all four reported positive findings for specific pesticides. In Sri Lanka, glyphosate applications associated with a highly increased risk for CKDu among male farmworkers, and an exposure-response for water intake from glyphosate polluted wells was observed in the only high explanation value study conducted in a CKDu epidemic area [95]. The herbicide glyphosate is a ubiquitously used nephrotoxic pesticide, including on rice in Sri Lanka and on sugarcane in Mesoamerica. The findings of this study are in accordance with a previously launched hypothesis that glyphosate, a metal-chelating agent, forms glyphosate-metal complexes in the presence of hard water and that the intake of such water could produce kidney damage [44, 101]. A very small study also examined urinary levels of many different metals and glyphosate in endemic CKDu cases (*n* = 10) and endemic (*n* = 10) and non-endemic (*n* = 10) healthy controls [101]. Levels were higher in both cases and controls in the CKDu area as compared to the area without CKDu, but the higher levels in cases in the endemic area were compatible with leakage into urine due to renal damage (Gerd Sällsten, University of Gothenburg, personal communication). In addition, glyphosate was not identified as a risk factor in studies in the USA [97], El Salvador [94] and Nicaragua [99]. According to Jayasumana et al. [101], the time of appearance of the epidemic in Sri Lanka coincides with the introduction and subsequent widespread use of this herbicide in Sri Lanka. However, in Central America there are differences in timing between the use of glyphosate and the surge of the CKDu epidemic. Precisely, although glyphosate is aerially sprayed since the 1990s as a maturation agent in sugarcane fields situated in areas where most CKDu cases occur, increased CKD mortality in the MeN-endemic area of Guanacaste in Costa Rica was observed as early as in the 1970s, at least a decade before the introduction of glyphosate on the market [12]. Thus, as of today, glyphosate can be considered as a potential risk factor for CKDu in Sri Lanka, but not in Mesoamerica.

Although the findings of the other three studies with high explanation value contribute to evidence of associations between various types of pesticides and CKD, they cannot be generalized to explain the CKDu epidemics in other regions [75, 76, 96, 97]. Regarding the study in urban Delhi, the associations between CKDu stage ≥3 and dietary or environmental exposures to OC insecticides [75, 76] do not exhibit differences in CKDu occurrence beween men and women. Exposure to OC alone would neither explain the CKDu epidemics in Central America and Sri Lanka, mainly because the clear male predominance is not in line with overall environmental OC pesticide exposures. OCs have been widely used worldwide against vector born diseases and, in Central America, also intensively in cotton cultivation during the 1970s [102], including in several of the regions of El Salvador and Nicaragua with current CKDu epidemics. OCs were banned or severely restricted since the 1980s [103], but there are stockpiles of obsolete pesticides in controlled and uncontrolled sites that may contaminate water and soil and eventually lead to human exposures. However, the only Central American location with co-occurrence of identified environmental pesticide contamination and excess CKDu cases in both male and female inhabitants is Las Brisas in El Salvador [71, 72].

The US cohort study of licensed applicators observed causal associations between ESRD and a considerable number of specific pesticidal agents as well as to repeated medical visits and hospitalization due to unspecified pesticide use [97]. Most interesting is the association with paraquat, also implicated in ESRD among the wives of the applicators [96]. Paraquat is a widely used herbicide, including in the CKDu epidemic regions around the world, and its acute nephrotoxicy is well-known. The positive results from the USA cohorts [96, 97] raise questions about much overlooked nephrotoxic effects of different pesticides, not surprisingly since the kidney is an excretory organ of toxins, and this should be further explored in other settings. It seems feasible that the increased risk of ESRD related to paraquat use and medical conditions from pesticide exposures is a consequence of episodes of clinical or subclinical AKI caused by nephrotoxic pesticides. Noteworthy, clinical AKI is associated with development of CKD later in life [104].

Of note is that only six studies (7 articles), in five countries, specified pesticidal agents [75, 76, 94–97, 99]. Each study reported different associations or no-associations, except for paraquat which was associated with ESRD both among the licensed applicators and their wives. One possible interpretation of the incongruent pattern in different regions could be that different sets of contributing causes, including different pesticides, trigger the occurrence of the same disease in different regions. However, currently there is no reasonable evidence to sustain this hypothesis.

The clear predominance of CKDu among males in agricultural sectors of both Mesoamerica, Sri Lanka and India allows commenting about the occupational versus environmental nature of the epidemics. Male predominance may be a consequence of occupational exposures that are related to gender differences, such as pesticide mixing and spraying or strenuous work done mostly by men, or there may be a biological difference between sexes responding to a toxic or physical insult, or both. Relatively few studies have explored occupational differences more in depth through stratified analyses by sex. In El Salvador, CKD was much more prevalent among males on the community level, but women who had worked in sugarcane and cotton plantations were also at increased risk for CKD just as their male colleagues, which suggests that the gender differences are in fact attributable to occupational exposures and not to sex differences [78].

Data examined at the time of the First and Second International Workshops on Mesoamerican Nephropathy in 2012 and 2015, respectively, led to insights that MeN is an occupational disease [2, 9]. The Consortium on the Epidemic of Nephropathy in Central America and Mexico (CENCAM) issued a statement that occupational heat stress is a likely key factor in the MeN epidemic and that pesticides is one of the risk factors that need to be investigated further, both a potential etiologic role and a possible role in disease progression [105]. It has been pointed out that heat exposure alone likely does not explain the disease pattern, and a ‘heat-plus’ hypothesis has been proposed [106]. On the other hand, Jayasumana et al. [28] questioned why in other regions with similar climatic conditions, there are no CKDu epidemics or, conversely, why CKDu occurs among people assumedly not exposed to extreme working conditions. Occupational pesticide and heat exposures co-occur in agricultural settings but no studies have looked yet into potential interactions between pesticides and heat stress, although a combined impact of these two separate factors seems plausible, as primary causal factors as well as in disease progression. Additional to its own adverse effects on the kidney, heavy physical workload in intense heat may result in increased exposure to putative nephrotoxic agrochemicals, because of greater pulmonary ventilation leading to greater inhaled intake, as well as of increased doses absorbed through the skin due to dilatation of skin’s capillaries and pores. Further in-depth exploration of the various identified or hypothesized risk factors and their interactions could improve the understanding of a possible multi-causality in CKDu epidemics.

## Concluding remarks

This review found some evidence of associations between pesticides exposure and CKD or CKDu, more clearly in studies with stronger design and better exposure assessment. Although these findings add to the recognition that certain pesticides produce acute and chronic kidney damage in humans, there is no strong epidemiologic evidence that pesticides are the culprit of the CKDu epidemics in Mesoamerica, Sri Lanka and beyond. Glyphosate in Sri Lanka could be an exception, but no associations have been seen for this herbicide in other CKDu regions. For a specific pesticide to be a key cause of an epidemic of the magnitude seen in Mesoamerica, Sri Lanka and India, it must be present during prolonged time periods in a diversity of agricultural settings in multiple countries, while generating elevated and widespread occupational or environmental exposures. Such a pesticide has not been identified.

Yet, up to today, no research has been conducted in CKDu endemic areas with a strong design and examining the role of lifetime exposures to specific pesticides or chemical groups with similar toxicological actions, especially not in combination with heat exposure or other major risk factors. Therefore, a role of nephrotoxic agrochemicals in the etiology of CKDu and the extent of their contribution to the CKDu epidemic, if any, cannot be adequately evaluated based on currently available data. Given the diversity of pesticide use, such research is difficult and costly, but necessary to elucidate the role, if any, of agrochemicals in this epidemic. We recommend that any future pesticide research should be conducted with the best possible assessment of lifetime exposures to relevant specific pesticides and enough power to look at interactions with other risk factors, in particular heat stress.

## Additional files

Additional file 1: Key terms used in the search strategy. (DOCX 29 kb)

Additional file 2: Table S1. Details of studies from Mesoamerica, Sri Lanka and other countries assessing the role of pesticides in chronic kidney disease. (DOCX 75 kb)

## Abbreviations

AChE: Acetylcholinesterase
ACR: Albumin creatinine ratio
AHS: Agricultural Health Study
AKI: Acute kidney injury
ANOVA: Analysis of variance
BUN: Blood urea nitrogen
CENCAM: Consortium on the Epidemic of Nephropathy in Central America and Mexico
CI: Confidence interval
CKD: Chronic kidney disease
CKDu: Chronic Kidney Disease of unknown etiology
CRF: Chronic renal failure
DB: Diabetes
DDE: Dichlorodiphenyldichloroethylene
DW: Drinking water
eGFR: Estimated glomerular filtration rate
ESRD: End-stage renal disease
F: Female
GST: Glutathione-S-transferase
HCH: Hexachlorocyclohexane
HT: Hypertension
IL-18: Interleukin-18
M: Male
MeN: Mesoamerican nephropathy
MVLR: Multivariate logistic regression
NAG: N-acetyl-β-o-glucosaminidase
NGAL: Neutrophil gelatinase-associated lipocalin
NSAID: Nonsteroidal anti-inflammatory drugs
OC: Organochlorine
OP: Organophosphate
OR: Odds ratio
PAHO: Pan American Health Organization
SCr: Serum creatinine
USA: United States of America
