# Erratum to: Pesticide exposures and chronic kidney disease of unknown etiology: an epidemiologic review

**DOI:** 10.1186/s12940-017-0274-9

**Published:** 2017-06-20

**Authors:** Mathieu Valcke, Marie-Eve Levasseur, Agnes Soares da Silva, Catharina Wesseling

**Affiliations:** 1 WHO-PAHO Collaborating Centre on Environmental and Occupational Health Impact Assessment and Surveillance INSPQ-CHUQ-DSPQ, 945, Avenue Wolfe, Québec, G1V 5B3 Canada; 2 Department of Environmental and Occupational Health, School of Public Health, Université de Montréal, C.P. 6128 Succursale Centre-Ville, Montreal, H3C 3J7 Canada; 3 Pan American Health Organization (PAHO), 525 Twenty-third Street, N.W, Washington, DC 20037 USA; 4 Department of Occupational Medicine, Institute of Environmental Medicine (IMM), Karolinska Institutet, 171 77 Stockholm, SE Sweden

## Erratum

This article [1] has been updated to correct the copyright information. The article was originally published with the below copyright statement:

*© The Author(s). 2017*

*Open Access This article is distributed under the terms of the Creative Commons Attribution 4.0*

*International License (* *http://creativecommons.org/licenses/by/4.0/* *), which permits unrestricted use, distribution, and reproduction in any medium, provided you give appropriate credit to the original author(s) and the source, provide a link to the Creative Commons license, and indicate if changes were made. The Creative Commons Public Domain Dedication waiver (* *http://creativecommons.org/publicdomain/zero/1.0/* *) applies to the data made available in this article, unless otherwise stated.*

The correct copyright statement is shown below and in the original article:

*© Pan American Health Organization. 2017*

*Open Access. This article is distributed under the terms of the Creative Commons Attribution IGO License (* *http://creativecommons.org/licenses/by/3.0/igo/legalcode* *), which permits unrestricted use, distribution, and reproduction in any medium, provided you give appropriate credit to the original author(s) and the source, provide a link to the Creative Commons license, and indicate if changes were made. In any reproduction of this article there should not be any suggestion that PAHO or this article endorse any specific organization or products. The use of the PAHO logo is not permitted. This notice should be preserved along with the article's original URL.*
