# Supplementary material for: Pesticide exposures and chronic kidney disease of unknown etiology: an epidemiologic review
Source: Environ Health. 2017 May 23;16:49. doi: 10.1186/s12940-017-0254-0 (PMC5442867; doi:10.1186/s12940-017-0254-0)
Supplement: Supplementary file 1 — Key terms used in the search strategy. (DOCX 29 kb) [file 12940_2017_254_MOESM1_ESM.docx]

**Additional file 1**

**SEARCH STRATEGY**

**Key terms used in the search strategy**

**PubMed (searched 2014/05/06)**

**Query 1:**

chronic renal disease*[tw] OR chronic renal disorder*[tw] OR chronic renal damage*[tw] OR chronic renal injur*[tw] OR chronic renal insufficienc*[tw] OR chronic renal failure*[tw] OR chronic renal dysfunction*[tw] OR chronic renal syndrome*[tw] OR chronic kidney disease*[tw] OR chronic kidney disorder*[tw] OR chronic kidney damage*[tw] OR chronic kidney injur*[tw] OR chronic kidney insufficienc*[tw] OR chronic kidney failure*[tw] OR chronic kidney dysfunction*[tw] OR chronic kidney syndrome*[tw] OR chronic nephrotic disease*[tw] OR chronic nephrotic damage*[tw] OR chronic nephrotic disorder*[tw] OR chronic nephrotic injur*[tw] OR chronic nephrotic insufficienc*[tw] OR chronic nephrotic failure*[tw] OR chronic nephrotic dysfunction*[tw] OR chronic nephrotic syndrome*[tw] OR OR renal chronic disease*[tw] OR renal chronic disorder*[tw] OR renal chronic damage*[tw] OR renal chronic injur*[tw] OR renal chronic insufficienc*[tw] OR renal chronic failure*[tw] OR renal chronic dysfunction*[tw] OR renal chronic syndrome*[tw] OR kidney chronic disease*[tw] OR kidney chronic disorder*[tw] OR kidney chronic damage*[tw] OR kidney chronic injur*[tw] OR kidney chronic insufficienc*[tw] OR kidney chronic failure*[tw] OR kidney chronic dysfunction*[tw] OR kidney chronic syndrome*[tw] OR nephrotic chronic disease*[tw] OR nephrotic chronic disorder*[tw] OR nephrotic chronic damage*[tw] OR nephrotic chronic injur*[tw] OR nephrotic chronic insufficienc*[tw] OR nephrotic chronic failure*[tw] OR nephrotic chronic dysfunction*[tw] OR nephrotic chronic syndrome*[tw] OR chronic nephropath*[tw] OR glomerular filtration rate*[tw] OR glomerulofiltration rate*[tw] OR glomerulo-filtration rate*[tw] OR kidney GFR[tw] OR glomerulus filtration rate*[tw] OR glomerular hyperfiltration rate*[tw] OR glomerular hyper-filtration rate*[tw] OR glomerulus hyperfiltration rate*[tw] OR glomerulus hyper-filtration rate*[tw] OR kidney function tests [tw] OR end-stage renal disease [tw]

AND

**Query 2:**

agrochemical*[tw] OR agro-chemical*[tw] OR agricultural chemical*[tw] OR fertilizer*[tw] OR fertiliser*[tw] OR insecticide*[tw] OR pesticide*[tw] OR chemosterilant*[tw] OR chemo-sterilant*[tw] OR chemical sterilant*[tw] OR fungicide*[tw] OR herbicide*[tw] OR fumigant*[tw] OR insect repellen*[tw] OR mosquito repellen*[tw] OR repel insect*[tw] OR repel mosquito*[tw] OR rodenticide*[tw] OR kidney disease risk factor*[tw]

AND

**Query 3:**

"2000"[Date - Publication] : "2014/04/30"[Date - Publication]

**= 83 results in PubMed**

**Lilacs** **(searched 2014/05/07)**

**Query 1:**

("chronic renal" OR "chronic kidney" OR "chronic nephrotic" OR "renal chronic" OR "kidney chronic" OR "nephrotic chronic") AND (disease* OR disorder* OR damage* OR injur* OR insufficienc* OR failure* OR dysfunction* OR syndrome*)

AND

**Query 2:**

agrochemical* OR agro-chemical* OR "agricultural chemical" OR "agricultural chemicals" OR fertilizer* OR fertiliser* OR insecticide* OR pesticide* OR chemosterilant* OR chemo-sterilant* OR "chemical sterilant" OR "chemical sterilants" OR fungicide* OR herbicide* OR fumigant* OR rodenticide*

**= 80 results in Lilacs = 70 results in English, Spanish and French**

**Embase, Medline, Total access collection, EBMR and Global Health (through OvidSP) for a total of 12 resources^[[1]](#footnote-1)^ selected (searched 2014/05/08)**

1. Concept 1 MeSH

exp renal insufficiency, chronic/ OR exp renal dialysis/ OR glomerular filtration rate/

2. Concept 1 EMTREE

chronic kidney failure/ OR exp renal replacement therapy/ OR glomerulus filtration rate/

3. Concept 1 langage naturel

((chronic ADJ2 (renal OR kidney OR nephrotic) ADJ5 (disease* OR disorder* OR damage* OR in jur* OR insufficienc* OR failure* OR dysfunction* OR syndrome* OR dialy* OR hemodialy* OR hemo-dialy* OR hemodiafiltration OR hemo-diafiltration OR hemodia-filtration)) OR (chronic ADJ2 nephro*) OR (("glomerul* filtration" OR glomerulofiltration OR glomurelo-filtration OR "glomerul* hyperfiltration" OR glomerulohyperfiltration OR glomurelo-hyperfiltration) ADJ rate*) OR "kidney GFR")

4. Concept 2 MeSH

exp agrochemicals/

5. Concept 2 EMTREE

exp agricultural chemical/ OR exp pesticide/

6. Concept 2 langage naturel

(agrochemical* OR agro-chemical* OR "agricultural chemical*" OR fertili#er* OR insecticide* OR pesticide* OR chemosterilant* OR chemo-sterilant* OR "chemical sterilant*" OR fungicide* OR herbicide* OR fumigant* OR ((insect* or mosquito*) ADJ3 repel*))

7. Concept 3 MeSH

exp epidemiologic studies/ OR evaluation studies/ OR prevalence/ OR incidence/ OR odds ratio/

8. Concept 3 EMTREE

exp epidemiology/ OR evaluation study/ OR exp risk/

9. Concept 3 langage naturel

(((epidemiologic* OR cross-sectional OR "disease frequency" OR prevalence OR case-control OR case-comparison OR case-compeer OR case-refer?ent OR case-base OR cohort OR concurrent OR incidence OR follow-up OR followup OR longitudinal OR evaluation OR descriptive) ADJ (study OR studies OR analys#s OR survey*)) OR "clinical report*" OR ((odds OR risk OR cross-product) ADJ1 (ratio* OR relative OR factor*)))

10. Concept 4 MeSH

(animals/ NOT (animals/ AND humans/))

11. Concept 4 EMTREE

(animal/ NOT (animal/ AND human/))

12. Concept 4 CAB Thesaurus

(animals/ NOT (animals/ AND man/))

13. Combining queries for concepts 1 and 2 and 3 not 4 in « langage contrôlé”

(1 OR 2) AND (4 OR 5) AND (7 OR 8) NOT (10 OR 11 OR 12)

14. Combining queries for concepts 1 and 2 and 3 in “langage naturel”

3 ADJ10 6 ADJ10 9

15. Combining the two requests (« langage contrôlé et naturel »)

13 OR 14

16. Limits (publication year)

limit 15 to yr=2000-2014

17. Deduping

..dedup 16

**= 776 results in OVID**

1. Périodiques électroniques - Forfait Total Access Collection, EBM Reviews - Cochrane Database of Systematic Reviews 2005 to February 2014, EBM Reviews - ACP Journal Club 1991 to March 2014, EBM Reviews - Database of Abstracts of Reviews of Effects 1st Quarter 2014, EBM Reviews - Cochrane Central Register of Controlled Trials January 2014, EBM Reviews - Cochrane Methodology Register 3rd Quarter 2012, EBM Reviews - Health Technology Assessment 1st Quarter 2014, EBM Reviews - NHS Economic Evaluation Database 1st Quarter 2014, Embase 1980 to 2014 Week 15, Ovid MEDLINE(R) 1946 to April Week 1 2014, Ovid MEDLINE(R) In-Process & Other Non-Indexed Citations April 14, 2014, Global Health 1973 to 2014 Week 14. [↑](#footnote-ref-1)
