# Supplementary material for: Pesticide exposures and chronic kidney disease of unknown etiology: an epidemiologic review
Source: Environ Health. 2017 May 23;16:49. doi: 10.1186/s12940-017-0254-0 (PMC5442867; doi:10.1186/s12940-017-0254-0)
Supplement: Supplementary file 2 — Details of studies from Mesoamerica, Sri Lanka and other countries assessing the role of pesticides in chronic kidney disease. (DOCX 75 kb) [file 12940_2017_254_MOESM2_ESM.docx]

Additional file 2: Table S1: Details of studies from Mesoamerica, Sri Lanka and other countries assessing the role of pesticides in chronic kidney disease. The explanation value refers to contribution of the study to clarify an association or no-association of pesticides with CKD or CKDu

| **Reference** | **Country &**  **area** | **Study design** | **Study population** | **Exposure assessment** | **Case definition / outcomes** | **Statistical analyses** | **Main findings** | **Authors’ conclusions**  **(in relation to pesticides)** | **Validity comments** | **Observed associations**  **---------------**  **Explanation value for CKD*** |
| --- | --- | --- | --- | --- | --- | --- | --- | --- | --- | --- |
| **Mesoamerica** | | | | | | | | | | |
| 1. Rugama, 2001 [85] | Nicaragua  University Hospital León, León Department | Retrospective hospital-based case-control | CKDu epidemic area  165 cases (125 M / 40 F): all hospital admissions with diagnosis of CKD during the year 2000  334 controls (152 M / 182 F): hospital admissions, randomly selected from all non-CKD admissions on the same day as cases | Data extracted from clinical records  Pesticide exposed yes/no | Diagnosis of CKD at hospital admission | Multivariate logistic regression model with pesticides, hot occupation, sex, urban vs rural, NSAIDs, and aminoglycoside antibiotics | OR pesticide exposure = 5.5 [95% CI 2.8 – 10.7] | Persons exposed to pesticides have greater risk for CKD than unexposed persons | Master thesis  Unquantified and nonspecific measure of pesticide exposure (medical annotations in clinical records)  Completeness and accuracy of pesticide exposure data in clinical records not addressed  Confounding by age: cases on average 10 years older than controls, without adjustment in analyses  Traditional risk factors not considered | Positive association between pesticide exposure and ESRD  --------------------  Explanation value low |
| 2. Trabanino *et al.*, 2002 [80] | El Salvador  National reference hospital in San Salvador | Descriptive survey among ESRD patients | CKDu epidemic region  ESRD patients admitted to hospital between Nov 1999 and March 2000  -67 CKD with primary diagnosis  -135 with unknown cause (CKDu) | Questionnaire-based personal interview  -Current agricultural occupation yes/no  -History of frequent contact with pesticides yes/no | Comparison of the two types of CKD groups (with regard to pesticide use and other risk factors) | No statistical testing | CKDu patients are predominantly men from coastal areas and younger than CKD patients with primary diagnosis  Agricultural occupation: 21% of CKD with primary diagnosis vs 63% of CKDu patients  Pesticide exposure: 25% of CKD patients with primary diagnosis vs 73% of CKDu patients | Agricultural occupation and pesticide exposure more frequent among patients with CKDu than among patients with CKD with primary diagnosis  No conclusion about causality because of descriptive nature of the study | An exploratory study of cases that did not intend to study etiology | Descriptive study  ----------------------  Explanation value none |
| 3. Gracia-Trabanino *et al.*, 2005 [86] | El Salvador  Communities in Jiquilisco Municipality (Pacific coast) and in Sesorí Municipality (non-coastal highlands at 500 masl) | Cross-sectional survey | CKDu epidemic region  First screening phase: random adult male (volunteer) sample from  -coastal area, N = 292, 35% of total men  -area at 500 masl, N = 62, 16% of total men  ---------------  Second phase only at the coast:  Further examination of 80 subjects out of the 133 who tested positive for proteinuria. Of these: 37 CKD cases: 12 CKD with primary diagnosis and 23 with CKDu | Questionnaire based personal interview  -Agricultural occupation yes/no  -Past exposure to pesticides yes/no | Screening:  Proteinuria >15 mg/L  --------------  Second phase:  CKD: SCr >1.5 mg/dL | Screening:  Prevalence and multivariate logistic regression with adjustment for unspecified predictors  ----------------  Second phase:  Chi-square tests | Screening phase:  -Proteinuria: coast 45.5% vs altitude 12.9% (82% proteinuria ≤30 mg/L)  Multivariate  OR agricultural occupation = 1.62 [0.75-3.49]  OR pesticide exposure 0.79 [0.42-3.47]  -----------------------------  Second phase:  Agricultural work not associated with CKD (p=0.47)  Pesticide exposures not associated with CKD (p=0.49) | Agricultural work or pesticide exposure not associated with proteinuria or with CKD | Unquantified and nonspecific measure of pesticide exposure  Recall bias unlikely with subjects unaware of disease status  Proteinuria as screening method has low sensitivity for detecting CKDu cases  Unclear adjustment for confounders  Loss of 40% of subjects with proteinuria for determination of CKD in second phase | No association of pesticide exposure with proteinuria (first phase) and CKD (SCr >1.5 mg/dL) (second phase)  -----------------------  Explanation value low |
| 4. Torres-Lacourt *et al.*, 2008 [88] | Nicaragua  La Isla & Candelaria, Chichigalpa Municipality, Chinandega Department | Cross-sectional population-based survey | CKDu epidemic region  Random sample of adults aged 20-60 in two different rural communities (known for high risk of CKDu), about 45% of eligible population, N= 377 (129 M / 248 F) | Face to face questionnaire:  -Mixing or applying pesticides yes/no  -Previous pesticide intoxication yes/no | Reversible CKD (stage 1-2) and irreversible CKD (stage 3-5)  (Q/DOKI norms National Kidney Council in 2008) | Prevalence of CKD by current occupation  Logistic regression with unadjusted ORs of risk factors for CKD stages 1-2 and 3-5 | Overall 42.4% of population with CKD stage 1-5:  -Stage 1-2: men 36.4%; women 27.5%  -Stage 3-5: men 31.0%; women 4.2%  Prevalence of CKD stage 3-5 among men between 40-60 yrs is 42.6% (51.7% between age 40-49 and 34.4% between age 50-60). Among active agricultural workers 27% had CKD stage 3-5.  ORs of CKD stage 3-5 in La Isla and Candelaria, respectively:  -current agricultural occupation 1.87 [0.88-3.99] and 2.68 [1.12-6.39]  -mixing/applying pesticides 2.11 [0.99-4.5] and 4.80 [2.33-9.89];  -previous pesticide intoxication 1.22 [0.32-4.67] and 1.19 [0.31-4.59] | The study found a relationship with labor-related factors of agricultural work and exposure to pesticides | Not peer reviewed university report  Unquantified and nonspecific pesticide exposure measure  Some recall bias possible with some subjects being aware of disease status  No control of confounding  The two communities were similar and pooled data analyses would have made the study stronger. | Positive association between pesticide mixing/applying and CKD stage 3-5  No association with previous pesticide intoxication  ----------------------  Explanation value low |
| 5. Torres *et al.*, 2010 [77] | Nicaragua | Cross-sectional population-based survey | Both CKDu epidemic and non-CKDu regions  1096 subjects (479 M, 617 F) of age 20-60 in five communities. | Main economic activity in the village, including agriculture-related ones; current occupation | SCr > 1.2 mg/dL (♂) and 0.9 mg/dL (♀) | Multivariate logistic regression | Adjusted OR (MVLR) of high SCr for living in a banana / sugarcane village were 3.39 [1.67– 6.91] (♂) and 2.42 [95% CI 0.79–2.44] (♀) | None | Pesticides or fertilizers were not specifically addressed | No assessment of pesticide associations  ----------------------  Explanation value none |
| 6. Sanoff *et al.*, 2010 [89] | Nicaragua  Nine municipalities  in León and Chinandega Departments | Screening program with nested case-control analysis | CKDu epidemic region  Screening of 997 volunteers, age > 18 (848 M / 149 F)  -----------------  Nested case-control analyses among male participants, 112 cases, 222 controls | Face to face questionnaire  -Agricultural field labor  -Work with or exposure to pesticides yes/no  Further detailed in quartiles of years of pesticide use: 0-2; 2-8; 8-13 and >13. | Screening:  eGFr <60 ml/min/1.73m^2^ vs ≥60 ml  --------------------  Nested case control:  Cases: eGFR <60 ml/min/1.73m^2^  Controls: eGFr ≥80 ml | Screening:  Chi-square tests and logistic regressions: ORs adjusted for age, sex, diabetes, hypertension, family history CKD and BM  ------------------  Nested case-control: Multivariate logistic regressions with same adjustments as in screening study | Screening:  Pesticides p< 0.0001 (univariate)  OR agricultural field labor 2.48 [1.59. 3.89]  OR pesticides 1.38 [0.90, 2.11]  ----------------------------  Nested case-control:  OR agricultural field labor = 2.38 [1.44, 3.93]  OR pesticides = 1.57 [0.97; 2.55] | The authors emphasize the association with agricultural field work and omit in their conclusions pesticides for not reaching statistical significance at the p<0.05 level. | Volunteers: possible selection bias  Unquantified and nonspecific measure of pesticide exposure (results for years of exposure not presented)  No information given to evaluate risk of recall bias  Control of confounding for traditional risk factors but not for occupational predictors in multivariate analyses | Weak positive association for pesticide exposure  -----------------------  Explanation value medium |
| 7. O’Donnell *et al.*, 2011 [90] | Nicaragua  Quezalguaque Municipality, León Department | Cross-sectional population-based survey with nested case-control analysis | CKDu epidemic region  Random sample of individuals aged ≥18 from 300 out of 1882 eligible households, N = 771 (298 M / 473 F)  ---------------  Nested case-control analysis:  98 cases, 221 controls from the same household | Face to face questionnaire  Agricultural work yes/no  Any pesticide exposure yes/no  Mixing or applying pesticides yes/no | CKD stage 3 or higher | Survey:  Prevalence  Case-control: Univariate logistic regressions and logistic regressions adjusted by age and sex | 13% of population age ≥18 has CKD, more frequent in young males at low altitude  Univariate / sex and age adjusted:  OR agricultural work = 2.09 [1.08, 4.05] / 1.00 [0.44, 2.27]  OR any pesticide exposure = 2.45 [1.31 – 4.57] / 1.85 [0.84, 4.07]  OR mixing or applying pesticides: 1.78 [1.09 – 2.91] / 1.32 [0.66-2.64] | The authors did not conclude any association of CKDu with pesticides for not reaching statistical significance. | Despite reported random selection, 61% in prevalence study were women.  Controls were not randomly selected, but from the same households as cases, sharing environments.  Unquantified and nonspecific measure of pesticide exposure  Recall bias unlikely with subjects unaware of disease status  Instead of adjusting for sex, stratification by sex could have shown more clearly potential work-related associations, including for pesticide exposures  Equivocal results with a positive association for any pesticide exposure and no association for an indicator of higher exposures( mixing and spraying) | Positive association between CKD stage ≥3 and any pesticide exposure  No association with mixing or applying pesticides  -----------------------  Explanation value medium |
| 8. Orantes *et al.*, 2011 [91] | El Salvador  Communities in Bajo Lempa (Jiquilisco Municipality) | Community-based screening and  cross-sectional survey | CKDu epidemic region  Individuals from 375 families (88% of community members), age ≥18, N = 775 (343 M / 432 F) | Face to face questionnaire  -Agricultural occupation  -Contact with agrichemicals yes/no | CKD stage 1-5 and CKD stage 3-5  (defined by two determinations with a 3-months interval) | Prevalences  Logistic regression model including positive risk factors in univariate analyses (p<0.05): contact with agrochemicals, agricultural worker, age, sex, family history CKD, HTA, DM, obesity, overweight, hyperlipidemia, metabolic syndrome | Prevalence CKD stage 1-2: men 8.7%, women 7.6%  Prevalence of CKD stage 3-5: men 16.9%, women 4.1%  Adjusted ORs of CKD stage 1-5:  -OR agricultural occupation = 1.35 [0.63-2.88]  -OR contact with agrichemicals = 1.23 [0.66 – 2.31] | CKD predominated in agricultural workers exposed to non-traditional (occupational and toxic factors) and traditional risk factors (DM, HT, obesity and dyslipidemia) that may act synergistically. | Unquantified and nonspecific measure of pesticide exposure  Recall bias unlikely with subjects unaware of disease status  Only multivariate risk estimates for CKD stage 1-5  Possible over-adjustments in multivariate models (both pesticide exposure and agricultural occupation in model).  No sex-stratified analyses, possibly obscuring potential work-related associations, including for pesticide exposures | No association between contact with pesticides and CKD stage 1-5  -----------------------  Explanation value medium |
| 9. Payán-Rentería *et al.*, 2012 [83] | Mexico  Nextipac, State of Jalisco | Cross-sectional comparison of an exposed and an unexposed group | Non-CKDu region  25 male farmworkers who sprayed pesticide during last season vs 21 unexposed (undefined) workers  Exclusion criteria: not living in Nextipac and any pathology that could interfere with results interpretation | Pesticide exposed versus not pesticide exposed  Average time of pesticide use 19 years; 4-5 pesticide applications in a 5-month period. Most used pesticides: OPs (chlorpyrifos, terbufos, methamidophos), carbofuran, atrazine, toxaphene  20% of exposed had an acute poisoning during the season | For kidney function assessment variable of interest is serum creatinine  [The main outcomes of this study were AChE, lipid peroxidation, and DNA circulating in serum] | Unclear: comparison between groups with cluster multivariate analysis, not further defined and not apparent from the reported results | Mean and range of SCr non-significantly higher in exposed  SCr exposed 1.21 (range 0.9 – 1.5) vs 0.98 (range 0.8 – 1.1) among unexposed (P=0.242) | Although the results of the tests were within normal limits, the exposed farm workers showed a tendency to be worse than the workers from the unexposed group | Inadequate description of methodology  No information about selection of study groups and small study population  Unquantified and nonspecific measure of pesticide exposure; results for specific pesticides and years of exposures not presented  No control of confounding | Higher non-significant SCr among pesticide exposed farmers  -----------------------  Explanation value none |
| 10. Peraza *et al.*, 2012 [78] | El Salvador | Cross-sectional population-based survey | Both CKDu epidemic regions and non-CKDu regions  664 subjects (256 M / 408 F) of age 20-60 in five communities. | Face to face questionnaire  Type of agricultural work: lowlands *vs* highlands; plantation *vs* small scale. | SCr > 1.2 mg/dL (♂) and 0.9 mg/dL (♀) | χ2 tests and multivariate logistic regression | Prevalence ratio of 2.6 for men ever vs never worked in agriculture (p = 0.04,).  Adjusted OR of high SCr for 10-yr increases in work on sugarcane or cotton cultivation 3.1 [2.0 – 2.5] (♂) and 2.3 [1.4 – 3.7] (♀). | Pesticides or fertilizers were not specifically addressed | Pesticides or fertilizers were not specifically addressed | No assessment of pesticide associations  ----------------------  Explanation value none |
| 11. Laux *et al.*, 2012 [93] | Nicaragua  El Roblar, a coffee growing village at 1,000 masl in Matagalpa Department | Cross-sectional community-based survey | Non-CKDu region  All adults aged 20-60 in the village, N = 267 (120 M / 167 F) | Face to face questionnaire:  Work with pesticides yes/no | CKD stages and proteinuria | Prevalences  Univariate and multivariate logistic regressions (proteinuria) adjusted by age, sex, BMI, HTA, DM | Prevalence CKD stages 1-2: men 27.5%, women 21.1%; stage 3 men 0%, women 1.2%. Prevalence proteinuria: men 27.5%, women 21.8% (p=0.02).  77% of the 66 CKD cases present with proteinuria: OR for work with pesticides: 1.09 [0.60-1.98]. | The study cannot make conclusions about pesticides and CKD, but the kind of exposure experienced by this population is not singularly sufficient to cause any increased prevalence of CKD. | Unquantified and nonspecific measure of pesticide exposure  Not possible to stratify by sex for exposure of interest, since 93% of men worked in agriculture and 98% used pesticides.  CKD stages 1 and 2 were proteinuric cases. The prevalence of CKD stage 3 was less than 1% (2 cases), and both in women. | The study addressed CKD with traditional causes since CKDu was not present in this non-endemic area  No association between work with pesticides and proteinuria  -----------------------  Explanation value medium |
| 12. Mejía *et al.*, 2014 [81] | El Salvador  Three communities in Bajo Lempa in Jiquilisco with cultivation of corn, beans, orchards, and production of sugarcane | Descriptive of pesticide exposure among CKDu cases | CKDu epidemic region  42 cases with confirmed CKDu (out of 51), with inclusion criteria to be a male farmer >18y and involved in pesticide management | Face to face questionnaire:  Past exposures to various types of pesticides and exposure conditions | No further definition of stages of CKDu of the cases | Descriptive statistics of variables related to pesticide handling  No statistical testing. | Most common pesticides used were 2,4-D, glyphosate, paraquat and several organophosphate insecticides  High prevalence of various inadequate pesticide use characteristics among the CKDu cases. | Farmers with CKDu have had high exposure to toxic pesticides due to the misuse in almost all stages of pesticide management; this implies non-occupational environmental hazards | A description of inadequate pesticide use among CKDu cases with a history of pesticide exposure | Descriptive study  ----------------------  Explanation value none |
| 13. Raines *et al.*, 2014 [45] | Nicaragua  Agricultural community in Chichigalpa | Cross-sectional population-based survey with nested case-control analysis | CKDu epidemic region  Survey: All individuals aged 15-69 (N=424, 166 M / 258 F)  Nested case-control:  78 cases (48 agricultural workers)  202 controls (69 agricultural workers) | Researcher-administered questionnaire:  Agricultural worker  Agricultural worker with lifetime days of:  -mixing pesticides  -applying pesticides  -working in areas  with ongoing or recent pesticide applications  Accidentally inhaling pesticides (not further specified)  Degree of personal protective equipment  Personal exposure route (face/lung/mucus membrane>skin>no exposure) | eGFR<60 ml/min/1.73m2 (using the CKD-EPI equation) and proteinuria  Case-control:  Cases eGFR <60 ml/min/1.73m2 Controls eGFR >90 ml  (Q/DOKI norms National Kidney Council) | Prevalence of eGFR<60 and proteinuria  Case – control:  Univariate and multiple logistic regression (age, sex, SBP, smoking)  Multivariate analyses restricted to agricultural workers adjusted for age and sex | eGFR<60 men 41.9%; women 9.8%  proteinuria ≥30mg/dL overall 15%  OR agricultural worker = 2.05 [0.61-6.90]  Subset agricultural workers:  - unadjusted lifetime days of mixing and lifetime days of applying pesticides not associated, p=0.13 and p=0.22 respectively.  Level of personal protective equipment not associated (p=0.35).  Personal exposure route among mixers and applicators (face/lung/mucus membrane>skin>no exposure) associated with eGFR<60, p=0.03 (type of analysis not specified).  Adj. OR past accidental inhalation of pesticides = 3.14 [1.12 – 8.78]. | Sugarcane cutting and harvesting is an important risk factor for development of CKD, which appears to be driven by a multifactorial combination of heat stress and concurrent sugar consumption, with possible contribution of pesticide exposure. | Relatively low response (62%)  Nonspecific measure of pesticide exposure, but with quantification of number of lifetime days  No analyses reported for most commonly used specific pesticides (2,4-D, glyphosate and paraquat)  The significant result for exposure route is not interpretable.  Strongest association for “Accidental past inhalation of pesticides” is based on an unclear exposure variable, whereas the main exposure measures do not show associations.  Some subjects in the studied communities may have been aware of disease status with possible recall bias for pesticide exposure | Vague variable of self-reported accidental inhalation of pesticides associated with decreased kidney function  No association with life-time days of pesticide use  -----------------------  Explanation value medium |
| 14. Vela *et al.*, 2014 [82] | El Salvador  Two distinct agricultural communities at 300 masl, El Jícaro (corn) in Usulután department and Dimas Rodríguez (subsistence farming) in San Salvador department | Descriptive study | CKDu epidemic region  Population age ≥ 15 (N=223, 111 M / 112 F) (91,4% response) | Face to face questionnaire:  Contact with agrichemicals yes/no | CKD stages 1-2 and CKD stages 1-5 | Prevalences of CKD indicators and risk factors  No statistical testing | CKD stage 1-2 much higher frequency CKD in El Jícaro than in Dimas Rodríguez (47.7% vs. 8.4%), but CKD stage 1-5 52.3% vs 47.4%  96.4% of men and 54.9% of women were farmworkers  94.5% of men contact with agrochemicals and 59.3% of women  Prevalence of CKD stages 1-5 was similar between farmworkers and non-farmworkers  Paraquat, 2,4-D, glyphosate and methyl parathion most often used | CKD prevalence is alarming in these communities, among both young and old, men and women, independently of occupation. | Practically all non-farmworkers were women.  Large differences of prevalences between CKD stages 1-2 and stages 1-5 between communities and sexes imply much higher frequency of CKD stage ≥ 3 in Dimas Rodríguez among women. Such distinct epidemiologic profiles for the communities were not explored in the light of agricultural work, pesticide use or other risk factors. | Descriptive study  ----------------------  Explanation value none |
| 15. VanDervort *et al.*, 2014 [79] | El Salvador | Ecological design | Both CKDu epidemic regions and non-CKDu regions  16,384 CKDu hospital admissions, incl. 8342 non diabetic ESRD cases. | Qualitative description of main pesticides used in different crops and current GIS-based % area of crop cultivation per municipality | CKDu, ESRD | Geographically-weighted regression models | Highest predictive model for hospital admissions due to CKDu (Rp^2^ = 0.80) and ESRD (Rp^2^ = 0.52) obtained for a combination of percent area of sugarcane, cotton and corn cultivation. | CKDu may arise from proximity to agriculture to which agrochemicals are applied, especially in sugarcane cultivation | Pesticides or fertilizers were not specifically addressed  Ecological fallacy bias, possibility of repeated hospital admissions and effect of high temperature in sugarcane not addressed. | Ecological study for hypothesis generation  ----------------------  Explanation value none |
| 16a. Orantes *et al.*, 2014 [71] | El Salvador  11 communities in 3 distinct regions: Bajo Lempa (BL, agriculture), Guayapa Abajo (GA, sugarcane) and Las Brisas (LB, semi-urban with dismantled storage depot of toxaphene and documented well water contamination) | Cross-sectional population-based survey | CKDu epidemic regions  Population age ≥ 18y (N=2388, 976 M / 1412 F) | Face to face questionnaire  Agricultural worker yes/no  Contact with agrochemicals yes/no  Contact with specific agrochemicals, most frequently methyl-parathion, methamidophos, phoxim, paraquat and 2,4-D | CKD stages 1-5  CKD defined as persistence of renal damage markers for ≥3 months  or  eGFR <60 ml/min/1.73 m^2^ | Prevalences of CKD and risk factors by subgroups of regions and sex  Multivariate logistic regression with risk factors that were significant in univariate analyses | Agricultural occupation associated with CKD stages 1-5 in BL, OR=2.5 [1.5-3.7], and in GA, OR 3.1 [1.5-6.5], but not in LB  No results reported for contact with agrochemicals in the agricultural communities BL and GA  Association of exposure to methyl-parathion with CKD in LB, OR 2.6 [1.2– 5.4] | The results of this study reinforce the hypothesis emerging from other research suggesting a new nephropathy, which could be called agricultural nephropathy | Unclear measure of pesticide exposure; no description of the methods used to assess exposure to specific pesticides  Differences of CKD patterns by sex and occupation between the agricultural and suburban region unexplored in the analyses | A positive association with agricultural work in the two farming communities  Positive association with methyl-parathion in the suburban community is not interpretable  ----------------------  Explanation value none |
| 16b. Orantes-Navarro *et al.*, 2015 [72] | The same study as Orantes et al, 2014 (see above) focusing on women | Idem | 1412 women aged ≥ 18 years | Idem | Idem | Descriptive statistics, prevalences.  No statistical testing | 15.2% of women were agricultural workers  33.1% had contact with agrichemicals  Prevalences CKD stages 1-5:  9.5% in BL, 13.9% in GA and 21.5 in LB  CKD stages 3-5:  3.2% in BL, 6.3% in GA and 13.4% in LB  No differences between agricultural and non-agricultural occupations among women | CKD in women in Salvadoran agricultural communities affects disadvantaged populations, with traditional (DM, HT, obesity) and non-traditional causes (environmental and occupational exposure to toxic agents and inadequate and unsafe working condition) playing roles | Descriptive analyses only | Descriptive report of subgroup of 16a  ----------------------  Explanation value none |
| 17. Herrera *et al.*, 2014 [17] | El Salvador  Same source population as in Orantes et al, 2014, plus Chalatenango | Case series | CKDu epidemic region  46 CKD cases, aged 18-59 according to inclusion and exclusion criteria (response rate 45%) | Personal interview by sociologist with tailored questionnaire that covered structural and intermediate  determinants  Farming occupation  Contact with agrochemicals | CKD stage 2, 3a and 3b, without known cause, or with HT or DM but without proteinuria, with normal fundoscopic exam and no structural abnormalities | No statistical testing, no comparison group | 89.1% farmers  95.7% contact with agrichemicals  Inadequate handling of pesticides of among CKD farmer patients | CKDu in Salvadoran farming communities is associated with  social and working conditions | Descriptive primarily clinical study | Descriptive study  ----------------------  Explanation value none |
| 18a. Laws *et al.*, 2015 [73] | Nicaragua  Chinandega, northwestern Nicaragua | Cohort, over one 6-month sugarcane harvest season | CKDu epidemic region  284 Nicaraguan sugarcane workers (251M, 33F), age ≥18, performing seven distinct tasks during the entire harvest, including agrochemical applicators (N=29) | Job title  Field worker (including agrichemical applicators) vs non-field worker (reference group)  Agrichemical applicators vs factory workers (reference group) | eGFR (using the CKD-EPI equation) as continuous variable | T-tests and multivariate linear regressions (models with age, sex and years worked) for group differences at pre- and late-harvest:  Paired t-tests and multivariate regressions (covariates age, sex and years worked) for change over the harvest season | Field workers vs non-field workers:  Mean group difference pre-harvest:  -0.8 [-4.7, 3.1]  Mean group difference late harvest  -7.8 [-12.3, -3.2]  Mean change during harvest season  -6.9 (-10.6, -3.2)  Agrichemical applicator vs factory worker:  Mean group difference pre-harvest  + 1.6 (24.7, 7.9)  Mean group difference late harvest  -2.2 (-9.6, 5.2)  Mean change during harvest season  -3.8 (-9.9, 2.3) | Among the field workers, agrichemical applicators experienced the least decline in kidney function. Because these workers have the most direct contact with agrichemicals, this finding does not support the hypothesis that agrichemicals are a causal agent. | Important loss to follow-up  Job category is a better proxy for pesticide exposure than ‘ever pesticide use’ or ‘agricultural worker’. It remains, however, a qualitative non-specific exposure proxy of current pesticide exposure that can imply also other putative causal exposures. | No association between a job as pesticide applicator and loss of kidney function or markers of early kidney damage over a six-month spraying period  ----------------------  Explanation value medium |
| 18b. Laws *et al.*, 2016 [74] | Idem | Idem | Idem | Job as pesticide applicator | Biomarkers of kidney injury: NGAL, NAG, IL-18, ACR | Paired-T test, Spearman rank correlations  Multiple logistic regressions | Mean changes early injury markers for pesticide applicators during harvest season:  NGAL -0.1 µg/g (p=0.9)  NAG -0.12 µg/g (p=0.6)  IL-18 -1.2 ng/g (p=0.6)  ACR +0.3 mg/g (p=0.8) | No increase in biomarkers of kidney injury in relation with a job of pesticide application or manipulation. |  |  |
| 19. García-Trabanino *et al.*, 2015 [94] | El Salvador  3 regions: at the coast, at 265 masl and 400 masl | Occupational cross-sectional survey (for the purpose of pesticide related analyses) | CKDu epidemic region  189 sugarcane cutters, 89% men, many with subsistence farming activities | Face to face questionnaire  Pesticide use ever (89%)  Specific pesticides used ever: glyphosate (76%), paraquat (82%), 2,4-D (71%); OPs (76%), carbamates (35%), triazines (73%), pyrethroids (59%) | Reduced pre-shift kidney function: eGFR <60 ml/min/1.73 m^2^ | Logistic regression model with age, region, BMI, smoking, kidney stones, hypertension, NSAID use, diuretics, number of previous harvests, region, any use of pesticides / use of specific groups of pesticides | ‘Any use of pesticide ever’ was not a predictor of reduced eGFR.  Ever use of carbamate pesticides 74% among the workers with reduced eGFR versus 29% among remaining workers. Significant predictor of low eGFR, also in the multivariate model  No associations of low eGFR with other groups of pesticides. | Self-reported previous use of carbamates was more common among cases with reduced eGFR and should be addressed in larger studies of MeN. | Unquantified exposure measure of specific chemical groups  Recall bias unlikely with subjects unaware of disease status | Positive association between ever use of carbamates and low kidney function  No associations with other chemical groups of herbicides or insecticides  ----------------------  Explanation value medium |
| 20. Wesseling *et a*l., 2016 [99] | Nicaragua  León and Chinandega in northwestern Nicaragua | Occupational cross-sectional study | CKDu epidemic region  86 sugarcane cutters, 56 construction workers, 52 subsistence farmers | Face to face questionnaire  Cane cutters, construction workers and subsistence farmers, respectively:  History of pesticide use: 47%, 11%, 73%  Glyphosate: 20%, 0%, 4%  Paraquat: 9%, 4%, 25%  2,4-D: 23%, 0%, 10%  Chlorpyrifos: 0%, 0%, 23%  Cypermethrin: 19%, 4%, 43% | eGFR< 80 ml/min/1.73m^2^ (using the CKD-EPI equation) | Chi-square for differences between occupational groups  Pesticides were not included in multivariate models because of lack of association in bivariate models | Cane cutters, construction workers, subsistence farmers, respectively:  eGFR<80 16%, 9%, 2%  No differences in history of pesticide use and no differences for the specific pesticides between workers with eGFR<80 and eGFR≥80, both in the pooled population and in the subset of sugarcane cutters (p-values between 0.14 and 1.00) | Farmers had the highest pesticide use and the best kidney function of the three groups.  Pesticide exposures were not an independent risk factor for reduced kidney function. | Unquantified exposure measures of specific pesticides  Recall bias unlikely with subjects unaware of disease status | No association between a history of any pesticide use  No associations between use of specific nephrotoxic pesticides and reduced kidney function  ----------------------  Explanation value medium |
| **Sri Lanka** | | | | | | | | | | |
| 21. Peiris-John *et al*.*,* 2006 [87] | Sri Lanka  -Agricultural Anuradaphura in North-Central Province  -Colombo  -Uda Walawe  -Colombo suburb Moratuwa | A cross-sectional comparison of red blood cell AChE (acetylcholinesterase) levels in 4 groups: OP exposed and unexposed CRF patients, and OP exposed and unexposed subjects without CRF | Both CKDu epidemic and non-CKDu regions  -23 OP exposed  CRF patients from Teaching Hospital in Anuradaphura (agricultural area)  -18 CRF patients from National Hospital, Colombo (urban non-farming)  -239 OP-exposed farmers without CRF from the Uda Walawe irrigation scheme (OP exposed)  -50 fishermen without CRF from Moratuwa | Differences in AChE (U/g) levels among the groups  (lower levels indicate higher OP exposures) | Chronic renal failure (CRF) is not defined | Unspecified tests for group differences.  All groups were compared to all each other and statistical significance was reported at the p<0.05 level | The order of AChE levels from lowest to highest:  1.Exposed farmers with CRF: 18.6 U/g  2.Unexposed CRF patients 26.6 U/g  3.Exposed farmers without CRF: 29.1 U/g  4.Unexposed fishermen without CRF: 32.6 U/g  Each group was significantly different from all other groups. | CRF patients (both exposed and unexposed) had lower levels of red blood cell AChE as compared to non-CRF exposed farmers and controls. | The description of methods and reporting and discussion of results are deficient.  OP exposure status was an assumption based on agricultural activities.  The selection of patients with CRF is not described  Study participants without CRF (OP exposed and unexposed) were participants in other studies. | Significant differences in AChE activity between each one of the four groups that follows both CRF and exposure status  ----------------------  Explanation value low |
| 22. Wanigasuriya *et al.*, 2007 [36] | Sri Lanka  Teaching Hospital in Anuradaphura | Hospital-based case – control (prevalent cases) | CKDu epidemic region  CKD cases: randomly selected among patients with CKD of unknown etiology (N=183; 136 M / 47 F)  Controls: randomly selected from among patients with hypertension, diabetes etc. without CRF (N=200, 139 M / 61 F), corresponding to same age group as cases (age 36-67) | Face to face interview:  -Agricultural occupation yes/no  -Pesticide handling yes/no  -Drinking-water source at home and in the field (well at home, pipe water, well in the field) | SCr >2.0 mg/dL | Independent sample t tests, chi-square tests, Fisher’s exact tests and odds ratios  Multivariate logistic regressions | Farming activities: males 86% cases and 57% controls; females 62% of cases and 56% controls. Pesticide use: males 72% cases and 47% controls; female 0% cases, 5% controls.  Risk factors in unadjusted analyses:  Males: OR farmer = 4.68 (2.50-8.82); OR pesticides = 2.94 (1.73-5.01), OR drinking from home well = 1.72 (0.92-3.22; OR pipe water = 0.19 [0.06-0.56], OR field well = 2.75 [0.96-8.22]  Females: OR farmers = 1.28 (0.55, 2.99); pesticides 0 cases, OR drinking from home well = 4.24 (1.51, 12.32); OR pipe water = 0.25 [0.05-1.04], OR field well = 1.31 [0.13-13.68]  Pesticide exposure was not retained as a contributing factor in the multivariate regression model, but familial aggregation was a strong risk factor. Well water may correlate with CRF in families. | Dismiss the hypothesis that long-term low-level (occupational or environmental) exposure to pesticides has an impact on the development of CRF.  CRF of unknown etiology in the North Central Province of Sri Lanka is probably due to an environmental factor, with the clustering of cases within families being due to a possible genetic predisposition to the potential environmental factor. | Selection of controls with HT and DM may not be adequate  25% of study population were women with CKDu but no history of pesticide use.  Unquantified and nonspecific measure of pesticide exposure  Possibility of recall bias cannot be evaluated with the given information.  Multivariate regression models not well described, and adjusted ORs for farming and pesticide use not presented (because p-values were non-significant)  Data on correlation of drinking well-water within families with CRF were not presented. | Association with drinking well-water at home, but not in the field  No associations with farming  No associations with pesticide use  ----------------------  Explanation value low |
| 23. Bandara *et al.*, 2008 [37] | Sri Lanka  Reservoirs in the North Central Province: Ullukkulama, Kumbichchankulama, Karapikkada, Alankulama, Thuruwila wewa  Patients and healthy subjects from Maddawachchiya and Girandurukotte | Descriptive study of contamination of environmental strata and human subjects | Both CKDu epidemic and non-CKDu regions  32 CRF cases not related to HT or DB and with proximal tubular sclerosis at biopsy  32 healthy subjects from the same area | Cadmium (Cd) concentrations in widely used fertilizers (triple super phosphate) and herbicide (bispyribac sodium) formulations  Levels of Cd (and other heavy metals) in water, soil, plants, milk, rice, fish  Urinary Cd (U-Cd) concentrations | Chronic renal failure | Not performed | 72 mg Cd / kg of triple superphosphate fertilizer  High levels of Cd in environmental strata  Estimate of weekly intake of Cd through rice, fish, lotus rhizomes and milk for age categories shows high total body burden: fish and rice staple 15.5-28.4 µg/kg body weight  U-Cd patients 7.58 ± 6.18 µg/g Cr  U-Cd healthy subjects 11.62 ± 8.45 µg/g Cr | The possible source of high Cd in reservoir water and sediments is Cd contaminated agrochemicals.  CRF in the North Central Province is a consequence of Cd contamination | The study describes Cd water and food contamination and Cd contamination of fertilizer and pesticide formulations as the likely source and cause of such contamination.  Urinary cadmium levels are higher in healthy study participants. | Descriptive study  ----------------------  Explanation value none |
| 24. Athuraliya *et al*, 2011 [19] | Sri Lanka  Three rural regions:  -Medawachchiya (North Central, high CKDu)  -Yatinuwara (Central, low CKDu)  -Hambantota (Southern, low CKDu) | Cross-sectional population-based survey with case –control type analyses | Both CKDu epidemic and non-CKDu regions  6153 (2889 M / 3264 F)  2600 Medawachchiya  708 Yatinuwara  2844 Hambantota  Aged > 19 years: randomly selected administrative units in the study regions, door to door recruitment in randomly selected households  109 CKDu patients in Medawachchiya (66 M, 43 F) | Face to face questionnaire  -Farmer yes/no  -Spraying or handling agrochemicals yes/no | Proteinuric-chronic kidney disease  Dipstick proteinuria ≥30 mg/dL on two out of three occasions over a 3-month period, and confirmed by sulfosalicylic acid test. | Prevalence for each study population adjusted for cluster effect.  Multivariate logistic regression (age, gender, farmer/non-farmer, direct exposure to agrochemicals, BMI, family history of CKD, diabetes, and hypertension) | Prevalence CKD (%CKDu): Medawachchiya 5.1% (84.0%); Yatinuwara 9.5 % (2.9%); Hambantota 2.3% (9.1%)  Entire study population:  Adj OR farmer 2.6 (1.9–3.4); adj OR agrochem. exposure 2.3 (1.4–3.9)  Medawachchiya  Adj OR farmer 2.1 (1.4–3.3); adj OR agrochem. exposure 1.1 (0.7–1.9)  Yatinuwara  Adj OR farmer 1.5 (0.5–3.9); adj OR agrochem. exposure 1.6 (0.8–3.2)  Hambantota  Adj OR farmer 1.6 (1.0–2.7); adj OR agrochem. exposure 5.6 (2.3–13.2) | Farming was associated to proteinuric CKD in Medawachchiya, whereas the combination of farming and exposure to agrochemicals in Hambantota, but in the latter region CKDu was infrequent. | Diagnosis based on proteinuria may have under-detected CKDu cases in the population.  Unquantified and nonspecific measure of pesticide exposure  Recall bias cannot be evaluated based on methods description  The overall increased risk for agrochemicals (RR 2.3) derives from high OR in Hambonta, a region almost without CKDu, whereas the other two regions (endemic and non-endemic CKDu showed no associations) | Pesticide use was not associated to proteinuric CKD in the CKDu endemic region  Pesticide use associated with CKD of known causes in one of two non-CKDu regions.  ----------------------  Explanation value medium |
| 25. Wanigasuriya *et al.*, 2011 [92] | Sri Lanka  Three divisional secretary areas in Anuradhapura (NCP):  -Medawachchiya (high CKDu)  -Padaviya (high CKDu)  Rajanganaya (relatively low CKDu) | Population-based cross-sectional survey with case –control type analyses | Both CKDu epidemic and low CKDu regions  886 (461 M / 425 F) household members aged ≥18 (60 randomly selected households from 3 randomly selected smaller administrative units (Grama Niladari with about 250 households each) in each secretary area. | Face to face questionnaire  -Farmer yes/no  -Involvement in pesticide spraying yes/no  -Previous pesticide poisoning  -Drinking-water source (well-water home, well-water field, pipe borne, stream) | Presence of micro-proteinuria by a two-step screening procedure  (first step detecting albumin between 10-200 mg/L and confirmation by second test) | Bivariate OR for prevalences of risk factors among subjects with and without micro-proteinuria.  Multivariate logistic regression with variables significant in bivariate analyses (model with HT, DM, urinary tract infection, drinking well water, smoking, pesticide spraying) | Bivariate analyses:  OR farmer = 1.38 [0.71, 2.70]  OR pesticide spraying = 1.01 [0.60, 1.72]  OR history pesticide poisoning = 0.67 [0.16, 2.84]  OR drinking water from well in the field = 1.79 [1.07, 3.01]  Multivariate model:  OR drinking well water in the field = 1.92 [1.04, 3.53]  OR pesticide spraying = 0.43 [0.21, 0.90]  Significant associations also with diabetes, hypertension and smoking | Drinking water from wells situated in the field is a predictor of early CKDu, which is possibly related to Cd pollution.  Protective effect of pesticide spraying may be due to pesticides being sprayed by subjects with young age, or by healthy worker effect. | Case finding is based on screening for proteinuria in a disease (CKDu) which is not characterized by proteinuria. Associations with traditional risk factors were not studied.  Unquantified and nonspecific measure of pesticide exposure  Potential for recall bias unknown based on methodologic description  Hypothesis and conclusions are partially based on the high environmental and urinary levels reported by Bandara *et al.* 2008. | Positive association with drinking well-water in the field  Pesticide use was not associated with micro-proteinuria in this population.  ----------------------  Explanation value medium |
| 26. Jayatilake *et al.*, 2013 [38] | Sri Lanka  3 endemic districts in North Central region:  -Anuradhapura  -Polonnaruwa  -Badulla  1 non-endemic district:  Hambantota | Cross-sectional population-based prevalence survey with case-control analyses.  Descriptive for the pesticide component | Both CKDu epidemic and non-CKDu regions  22 randomly selected villages (Grama Niladari) in 6 divisional secretary areas randomly selected from the 3 endemic areas. Random sample of 100 households per village, all members between age 15 and 70  N = 4777 (74% response)  CKDu: 733  Controls: 4044  Additional controls from the non-endemic area: n=250  Urinary pesticide concentrations CKDu cases N=57; Controls non-endemic area N=39 (controls are only mentioned in methods, no results reported)  Random sample of 495 for urinalyses of As, Cd and Pb and age- and sex-matched controls in the endemic (N=132) and non-endemic areas (N=250) | Face to face questionnaire:  -Farmer yes/no  -No protection from agrochemicals yes/no  Urinary concentrations of 11 pesticides or their metabolites in CKDu cases (and controls from the non-endemic area, but not reported): 2,4-D, 2,4,5-T, 2,4,5-trichlorophenol,  isopropoxyphenol, pentachlorophenol, 3,5,6-trichloropyridinol,  p-nitrophenol, 1-naphthol, 2-naphthol, glyphosate, aminomethylphosphonic acid (AMPA).  Analyses of phosphate fertilizer, pesticides and weedicides for As, Cd and Pb.  As, Cd, Pb, Se, Sr, Cr, Al in substrates of urine, hair, nails, serum in varying subsets of study participants; and in food (vegetables, fish, rice etc.), water (wells, reservoirs, pipes etc.), tobacco leaves, pasture, weeds, and soil. | CKDu: persistent albumin–creatinine  ratio (ACR) ≥30 mg/g (twice) + no past history of other causes of CKD  CKD stages 1-4 (based on CKD-EPI equation) | Logistic regressions of risk factors adjusted for age and sex.  Differences in levels of metals (and pesticides, results not presented) between CKD cases and controls and in endemic and non-endemic regions | Farmer: OR for CKDu (all stages) = 1.20 [1.01, 1.42]  Paddy farmer: OR = 0.73 [0.54, 1.00]  Well: OR = 0.97 [0.79, 1.20]  No protection from agrochemicals: OR = 1.01 [0.66, 1.55]  Pesticides detected in urine of CKDu cases:  2,4-D, 33%  3,5,6-trichloropyridinol, 70%  p-nitrophenol, 58%  1-naphthol, 100%  2-naphthol, 100%  Glyphosate, 65%  AMPA. 28%  Isopropoxyphenol, 2,4,5-trichlorphenol and pentachlorophenol below detection limits  Pesticides residues were above reference levels in 31.6% of those with CKDu  Results of metals in agrochemical formulations:  Mean U-Cd:  Individuals with CKDu 1.039 μg/g  Controls in the endemic area 0.646 μg/g (p<0.001)  Controls in non-endemic area 0.345 μg/g (p <0.05)  A significant dose–effect relationship between U-Cd and CKDu stage (P < 0.05) | Multiple agents may play a role in the pathogenesis of CKDu.  Chronic exposure of people in the endemic area to low levels of cadmium through the food chain and also to pesticides.  Significantly higher U-Cd in individuals with CKDu, and the dose–effect relationship between U-Cd and CKDu stages suggest that Cd exposure is a risk factor for the pathogenesis of CKDu. | OR for 'no protection from agrochemicals' is erroneous (subjects unexposed to pesticides were included in category of no protection).  Selection process of subjects for urinary pesticide residues was not described.  Results of urinary pesticide residues in 39 controls of non-endemic area were not presented.  In 31.6 % pesticide residues above given reference level: unclear what these reference levels stand for (no urinary tolerances exist for pesticide residues). | Descriptive results for pesticides  ----------------------  Explanation value none |
| 27. Jayasumana *et al.*, 2015 [95] | Sri Lanka  Padavi-Sripura, Eastern province, bordering NCP | Case-control (prevalent cases) | CKDu epidemic region  125 CKDu cases (89 M / 36 F): all CKDu patients, identified at medical clinic at Padavi-Sripura divisional hospital and confirmed by nephrologist  180 controls (98 M / 83 F): healthy individuals who came to the hospital for screening for CKDu during the study period (after excluding CKDu and other chronic diseases.) | Face to face questionnaire  -Usual occupation last 10 years  -Pesticide use, and specific pesticides up to 10 years back in time:  organophosphates,  paraquat, 2-methyl-4-chlorophenoxy-acetic acid (MCPA), glyphosate, bispyribac, carbofuran, mancozeb and other commonly used pesticides  -Drinking from wells and abandoned wells  -Analyses of glyphosate, metals, and hardness related parameters in water from wells (n=16), abandoned wells (n= 18), reservoirs (n=2), and well and pipe water from Colombo | CKDu  The criterion for CKD was not defined in the paper  Ministry of Health criteria used for inclusion of patients of unknown etiology:  -no history or treatment for diabetes or other pathologies underlying nephropathies  -normal glycosylated HbA1C<6.5%)  -BP ˂160/100 mmHg untreated or ˂140/90 mmHg on up to two antihypertensive agents | Bivariate and multivariate logistic regression (adjustment for age, sex, educational level, having a death among family members due to CKD and other exposure factors related to agrochemical use) | Bivariate regression:  -Farming: OR 3.12 [1.74-5.61]; pesticide application: OR 3.31 [2.04-5.36]; applying fertilizers: OR 2.37 [1.43-3.93]; drinking well water: OR 4.82 [2.27-10.24]; history of drinking from recently abandoned well OR 6.93 [I3.87-12.40]; male farmers (vs female farmers) OR 4.69 [1.06-20.69]; OPs: 1.77 [1.10-2.86]; paraquat 2.51 [1.56-4.04]; MCPA 1.80 [1.12-2.88]; glyphosate 4.33 [2.66-7.05]; bispyribac 2.00 [1.25-3.18]; carbofuran 1.47 [0.91-2.40]; mancozeb 1.94 [1.21-3.13]  Multivariate regression: OR drinking well water 2.52 [ 1.12-5.70]; OR history of drinking from recently abandoned well 5.43 [2.88-10.26]; OR pesticide application 2.34 [0.97- 5.57]; OR use of glyphosate 5.12 [2.33-11.26]  Water hardness: abandoned wells: very high; serving wells: moderate to hard; reservoir-pipeline: soft  Glyphosate concentration in water: abandoned well (median 3.2 µg/L) significantly higher than in serving wells [0.6 µg/L); reservoir waters: traces; Colombo pipe waters: not detected | The current study strongly supports the hypothesis that  CKDu in Sri Lanka is a drinking-water-related disease in farmers who have a history of spraying glyphosate.  Further studies should focus on abandoned drinking water  sources in areas with high prevalence of the disease and investigate the link between CKDu and glyphosate in particular, and heavy metals in drinking water | Cases were prevalent cases but likely comprised all CKDu cases in the Padavi-Sripura area at the time of the study  Controls came for screening of the same disease as the cases, but CKDu was investigated.  Exposure assessment for specific pesticides; although there was no quantification of their use. Recall bias possible for specific pesticides  A dose response was created by combining questionnaire data on source of drinking water and chemical analyses in the different types of drinking water. No recall bias possible with exposure data based on chemical analyses | Positive association with pesticide applications  Positive association with glyphosate use  Positive association with drinking well-water and with history of drinking water from abandoned wells (exposure – response with hardest water and highest glyphosate levels)  ----------------------  Explanation value high |
| 28. Rango *et al.* 2015 [84] | Sri Lanka  20 communities in seven districts endemic and non-endemic | Community-based case-control (prevalent cases) | CKDu epidemic region  26 CKDu cases (25 M / 1 F) identified with help of communities and health centers in endemic communities  108 controls (including 25 children age 10-18) (57 M, 51 F) randomly selected in endemic communities from remaining households (n=79) and in non-endemic communities (n=29) | Household questionnaire:  -Agrochemical use (fertilizer, herbicides, pesticides) yes/no  Concentrations of metals in urine (As, Cd, Pb, Mo, U) and others | Clinically confirmed CKDu diagnosis | Multivariate logistic regressions (covariates not clearly described) | Multivariate analyses:  Use of fertilizers higher among CKDu cases (p > 0.05 and ≤ 0.10)  Use of herbicides significantly higher in endemic communities (p ≤ 0.01) | CKDu not likely linked to presence of metals in drinking water. Based on available data, may act as cofactors with other agents.  Herbicides/fertilizers cannot be excluded. | Incomplete identification of cases in study area  Random selection of adults, with children added non-randomly.  Unquantified and nonspecific measure of exposure to pesticides and fertilizers | Inadequate methodology regarding study of pesticide associations  ----------------------  Explanation value none |
| **Other regions** | | | | | | | | | | |
| 29. Kamel & El-Minshawy 2010 [6] | Egypt  The 9 districts of El-Minia Governorate | Hospital-based case-control (prevalent cases) | Both CKDu epidemic and non-CKDu regions  Cases: ESRD patients without known cause (N=216) (141 M, 75 F) out of 800 ESRD patients presenting to 19 dialysis centers.  Controls: Random from patients in general hospitals with unrelated health problems (N=220) (152 M, 68 F)  Cases and controls matched for sex, age, smoking habits  Exclusion criteria: ESRD cases with known cause and controls with DM, HT and history of any renal disease. | Face to face questionnaire:  -Rural residency yes/no  -Drinking unsafe (non-pipe) water yes/no  -Farming occupation yes/no  -Pesticide exposure (storing pesticides at home or using pesticides at fields or selling or exposure by any mean) yes/no | ESRD of unknown cause | Test of proportions for bivariate analyses  Multivariate logistic regression | Residence in rural areas 76% vs 57% (p<0.001)  Drinking unsafe water 72% vs 48% (p<0.001)  Farmers 40% vs 20% (p<0.001)  Exposure to pesticides 52% vs 34% (p<0.001)  Multivariate analyses:  OR for living in rural area = 2.40 [1.59–3.61]  OR for exposure to pesticides 2.08 [1.42 – 3.06] | The high incidence of ESRD in El-Minia, Egypt, is probably due to an environmental factor. The clustering of patients within the families is possibly due to a genetic predisposition to the potential environmental factor.  ESRD with an unknown etiology attributed  to environmental factors such as drinking unsafe water, exposure to pesticides and using herbs for treatment. | Unknown if selection bias occurred  Unquantified and nonspecific measure of exposure  Recall bias not known based on methodologic description  Multivariate model not specified | Possible association with pesticide exposures  ----------------------  Explanation value low |
| 30a. Siddharth *et al.*, 2012 [75]  Note: this study is an interim report of Siddarth *et al.*, 2014 [76] | India  Dehli  Urban setting | Hospital-based case-control | Non-CKDu regions  Interim report of Siddarth et al, 2014  150 CKDu cases: at University College of Medical Sciences and GTB Hospital (77 M / 73 F)  96 controls: healthy age–sex-matched nonrelated persons accompanying patients at the renal clinic and staff of the hospital (51 M / 45 F)  Inclusion criterion: age 30-50  Exclusion criteria: cases on systemic steroids, diabetes and systemic or urinary tract infections; subjects occupationally exposed to pesticides or industrial chemicals, such as farmers | Blood levels of organochlorine pesticides (LOD 4 pg/mL), and total OC pesticide load (TPL)  Group differences in levels of specific OC  Among cases, tertiles of TPL levels | eGFR  CKDu with eGFR <60 ml/min/1.73 m^2^, > 3 months (MDRD)  Markers of oxidative stress | Spearman’s or Pearson’s correlation coefficients  Multivariate analyses: two-way ANOVA with Bonferroni analysis to adjust for the stages of CKD, age, sex, body mass index, lipid content, and individual pesticide levels in CKD patients | 9 OCs detected: α–HCH, β-HCH, γ-HCH, α-endosulfan, β-endosulfan, aldrin, dieldrin, p,p-‘DDT, p,p’-DDE.  Differences between groups: Significantly higher blood levels in cases for α–HCH, γ-HCH, total HCH, α-endosulfan, β-endosulfan, aldrin, p,p’-DDE, and TPL.  Among cases: Spearman correlations between eGFR and different pesticide analytes adjusted for age, sex, BMI, triglycerides, and total cholesterol between -0.07 and -0.23 (significant for γ-HCH, total HCH and aldrin). When adjusting additionally for levels of other analytes, association with eGFR only significant for aldrin. In addition, significant correlation between eGFR and TPL (r = -0.26).  Positive correlation between increasing levels of TPL and indicators of oxidative stress also when adjusting for stage of CKD. | The objective was to study relation between OCs, eGFR and oxidative stress  With decreasing eGFR, CKD patients tend to accumulate pesticides.  The accumulated total pesticides associated significantly with increased oxidative stress independent of eGFR.  The role of OCs in reduction of eGFR needs in vitro and prospective designs. | Inclusion of subjects with occupational pesticide exposures could have strengthened the study.  Correlation coefficients among cases only.  The coefficients are low (the highest 0.26), explaining only a small part of the variance in eGFR.  Higher OC blood levels among cases is possibly due to reverse causation | See below (30b) |
| 30b. Siddarth *et al.*, 2014 [76] | Idem | Idem | 279 CKDu cases: patients consulting at University College of Medical Sciences and GTB Hospital (140 M / 130 F)  270 controls: healthy age–sex-matched nonrelated hospital staff and persons accompanying patients at the renal clinic (140 M / 130 F)  Exclusion criteria: cases with known cause; subjects occupationally exposed to pesticides or industrial chemicals, such as farmers | Environmental OC exposures: blood levels of organochlorine pesticides and total pesticides in non-occupationally exposed subjects  Blood concentrations of OCPs were categorized  into three groups using control group tertile cutoff points  GSTT1(+)/GSTM1(+)  GSTT1(+)/GSTM1(-)  GSTT1(-) /GSTM1 (+)  GSTT1(-)/GSTM1(-) | eGFR  CKDu with eGFR <90 ml/min/1.73 m^2^, > 3 months (MDRD) | Differences between means with Mann–Whitney U test.  Multivariate logistic regression for tertiles of total OCs blood levels and CKD risk with adjustment for:  i) age, sex, BMI and total lipid;  ii) previous + all detected OCs  iii) previous + genotypes | Significant higher blood concentrations in cases for α–HCH, γ-HCH, total HCH, α-endosulfan, β-endosulfan, aldrin, p,p’-DDE, and total pesticides  Among cases: Spearman correlation coefficients between OCs and eGFR varied between -0.11 and -0.32. Significant adjusted correlations for γ-HCH (-0.24), total HCH (-0.27), aldrin (-0.24), and total pesticides (-0.32)  Significant associations with CKDu, 3rd vs 1st tertile of α-HCH (OR 2.64), γ-HCH (OR 3.12), total-HCH (OR 3.87), aldrin (OR 3.46), α-endosulfan (OR 3.63), β-endosulfan (OR 3.50), and p,p’-DDE (OR 2.70). When adjusting also for other analytes, significant for α-HCH, γ-HCH, total-HCH, aldrin, α-endosulfan, and β-endosulfan; and when adjusting also for genotype for α-HCH, total-HCH, and β-endosulfan. Total pesticides 3^rd^ vs 1^st^ tertile OR 2.73 [(1.46–9.47) and when adjusting also for genotype 2.32 (1.32–8.34)  Genotypes GSTM1(-) / GSTT1(-) vs GSTM1(+) / GSTT1(+) associated with CKDu, OR=1.81 [1.08–3.03]  CKD patients having either one null or two null genotypes accumulate majority of pesticides, whereas in healthy controls only some pesticides in the subset with both null genotypes. | Increased OC level in CKD patients is partially dependent on GSTM1/GSTT1 polymorphism and particularly GSTM1 (-) / GSTT1(-) genotype is more vulnerable.  Blood levels of OCs determined in CKD patients do not allow causal conclusions (i.e. inverse causation due to decreased excretion capacity) | Inclusion of subjects with occupational pesticide exposures could have strengthened study.  Correlation coefficients among cases only.  The coefficients are low (the highest 0.32), explaining only a small part of the variance in eGFR.  Genetic analyses partially resolve issue of reverse causation | Positive association of blood levels of OCs (from environmental exposures) with CKDu, mediated partially through genotype  ----------------------  Explanation value high |
| 31. Lebov et al, 2015 [96] | USA  Iowa and North-Carolina | Cohort  Recruitment between 1993–1997  Follow-up until December 2011 | Non-CKDu regions  Agricultural Health Study  Wives of licensed applicators  N= 31.142 with 98 ERSD cases between recruitment and end of follow-up identified through linkage with the US Renal Data System (USRDS) | Self-administered questionnaires or telephone interview:  -direct exposures (n=17,425): ordinal categories of intensity weighted lifetime use of any pesticide, 10 specific pesticides and 6 chemical classes  -Indirect pesticide exposures related to husband’s pesticide use among wives without personal use (n=13,717)  -Residential pesticide exposure through a variety of indicators including distance to field, hours in sun during growing season pre-recruitment, washing spraying clothes and others | ESRD identified through link with US Renal Data System | Cox proportional hazards models, adjusted for age | Highest category of cumulative lifetime-days of pesticide use in general vs never personal use: HR 4.22 [1.26-14.2]  Several specific pesticides high HR (>1.50) but non-significant and/or with very low number of exposed cases (<5) (alachlor, chlorymuron-ethyl, imazethapyr, petroleum oil, carbaryl)  No excess risk for glyphosate  Exposure-response trends for husband’s use of paraquat HR 1.99 [1.14-3.47] and butylate HR 1.71 [1.00-2.95]  No excess risk for indicators of residential exposures | ESRD may be associated with direct and/or indirect pesticide exposure among farm women | Cohort design with long follow up  Many comparisons and relatively few positive results.  Risk associated with paraquat is consistent with the results observed among male applicators (see study 32), but not butylate | Associations between ESRD and high cumulative pesticide use in women  No strong evidence for associations between indirect exposures to husband’s pesticide use, except for paraquat and ESRD  No evidence for associations between residential exposure to pesticides and ESRD  ----------------------  Explanation value high |
| 32. Lebov *et al.*, 2016 [97] | USA  Iowa and North-Carolina | Cohort  Recruitment between 1993–1997  Follow-up until December 2011 | Non-CKDu regions  Agricultural Health Study  55,580 male licensed pesticide applicators with 320 ERSD cases between recruitment and end of follow-up identified through linkage with the US Renal Data System (USRDS)  24,565 male applicators with additional pesticide information with 136 ERSD cases  Exclusion female applicators, age <18 yrs and ESRD cases diagnosed previous to recruitment | Exposure to 39 specific pesticides  Self-administered questionnaires:  –Enrollment questionnaire (55,480 applicators)  -Take home questionnaire (24,565 applicators) with additional data on pesticide use  -Ordinal categories of intensity-weighted lifetime use of 39 pesticides  -Doctor diagnosed poisoning and events of unusually high-level pesticide exposures  -Pesticide exposure resulting in a medical visit or hospitalization | ESRD identified through link with US Renal Data System | Cox proportional hazards models, adjusted for age and state  Covariates evaluated and not included in the final models: race, education, diabetes, BMI, hypertension  Sensitivity analyses  -correlated pesticides  -Lag time of 5 years post enrollment to address healthy worker effect | Exposure-response trends for the herbicides alachlor, atrazine, metolachlor, paraquat, and pendimethalin, and the insecticide permethrin  No excess risk for glyphosate use  Ever use of petroleum oil or imazethapyr significant HRs but no trends.  HR for the insecticides coumaphos, parathion and phorate (organophosphates), aldicarb (carbamate), chlordane (organochlorine), and for the fungicide metalaxyl were non-significantly elevated (HR >1.6)  More than one medical visit due to pesticide use HR=2.13 [1.17 - 3.89]  Hospitalization due to pesticide use HR=3.05 [1.67 to 5.58]  No increased risk with applicator report of doctor-diagnosed pesticide poisoning or unusually high personal pesticide exposure  Trend for increasing number of pesticide-related doctor visits (p=0.038)  In sensitivity analyses  -significant risk for coumaphos HR=1.81 [1.03 to 3.17]  -significant exposure-response trend for chlordane P=0.018  No associations for 25 of the 39 pesticides | The results suggest an association between ESRD and chronic exposure to specific pesticides.  Pesticide exposures resulting in medical visits may increase the risk of ESRD | An occupational cohort study with long follow up that assesses risk for ESRD from specific pesticides  Healthy worker effect was assessed which did not seem to be a problem  Support from experimental studies for most of the observed associations | Associations between ESRD and chronic exposure to a number of specific pesticides  Pesticide exposures resulting in medical visits or hospitalization associated with ESRD  ----------------------  Explanation value high |
| 33. Aroonvilairat *et al.*, 2015 [98] | Thailand  Nakhonpathom and Sakutsaphon provinces | Cross-sectional comparison of an exposed and an unexposed group | 64 farmers of orchids (30 M, 34 F) vs 60 non-farmers (33 M, 27 F) in the same districts, age 20-60  Exclusion criteria: immunologic conditions, pregnancy, lactation, diabetes | Non-CKDu regions  Work on orchid farm for at least 3 months  The main job of 88% of the farmers was spraying and mixing pesticides.  Most frequently used pesticides in the last three months: cypermethrin, abamectin, various carbamates, various organophosphates, captan, mancozeb, glyphosate and paraquat. | Difference in BUN and SCr levels (mg/dL continuous) | Mean difference by Student’s *t*-test; and differences in percentage of abnormal results by Chi-square and Fisher exact test | BUN (mg/dL) exposed 12.64 ±3.7 (3.7% abnormal) vs BUN unexposed 12.43 ± 2.9 (1.7% abnormal), p=0.76  SCr (mg/dL) exposed females 0.86 ± 0.11 (3.7% abnormal) vs unexposed females 0.82 ± 0.11 (2.9% abnormal), p=0.11  SCr exposed males 1.09 ± 0.11 (0% abnormal) vs unexposed males 1.09 ± 0.10 (0% abnormal), p=0.95 | No differences in kidney function between exposed and unexposed | Main objective was to evaluate immunologic parameters.  Scant description of selection of controls  Unquantified and nonspecific measure of exposure Multivariate model not specified  No multivariate analyses | No association between occupation in highly pesticide exposed farming and decreased kidney function  ----------------------  Explanation value low |
